# 2-(2-Phenylethyl)-4*H*-chromen-4-one Derivatives from the Resinous Wood of *Aquilaria sinensis* with Anti-Inflammatory Effects in LPS-Induced Macrophages

**DOI:** 10.3390/molecules23020289

**Published:** 2018-01-30

**Authors:** Sin-Ling Wang, Yun-Chen Tsai, Shu-Ling Fu, Ming-Jen Cheng, Mei-Ing Chung, Jih-Jung Chen

**Affiliations:** 1 School of Pharmacy, College of Pharmacy, Kaohsiung Medical University, Kaohsiung 807, Taiwan; s8332805@yahoo.com.tw; 2 Institute of Traditional Medicine, National Yang-Ming University, Taipei 112, Taiwan; tyc202006@gmail.com (Y.-C.T.); slfu@ym.edu.tw (S.-L.F.); 3 Bioresource Collection and Research Center (BCRC), Food Industry Research and Development Institute (FIRDI), Hsinchu 300, Taiwan; cmj@firdi.org.tw; 4 Faculty of Pharmacy, School of Pharmaceutical Sciences, National Yang-Ming University, Taipei 112, Taiwan; 5 Department of Medical Research, China Medical University Hospital, China Medical University, Taichung 404, Taiwan

**Keywords:** *Aquilaria sinensis*, Thymelaeaceae, resinous wood, structure elucidation, 2-(2-phenylethyl)-4*H*-chromen-4-one, anti-inflammatory activity

## Abstract

The resinous wood of *Aquilaria sinensis*, known as agarwood (*Chen Xiang* in Chinese), is traditionally used for the treatment of abdominal pain, vomiting, circulatory disorders, and dyspnea. Four new 2-(2-phenylethyl)-4*H*-chromen-4-one derivatives, namely 7-methoxy-2-[2-(4′-hydroxy-phenyl)ethyl]chromone (**1**), 7-hydroxy-2-[2-(4′-methoxyphenyl)ethyl]chromone (**2**), 5,6-dihydroxy- 2-[2-(3′-hydroxy-4′-methoxyphenyl)ethyl]chromone (**3**), and 6-hydroxy-5-methoxy-2-(2-phenyl-ethyl)chromone (**4**), have been isolated from the resinous wood of *A. sinensis*, together with nine known compounds. The structures of these compounds were determined through spectroscopic and MS analyses. Among the isolated compounds, neopetasan, 7-methoxy-2-(2-phenylethyl)-chromone, 6,7-dimethoxy-2-(2-phenylethyl)chromone, and 6,7-dimethoxy-2-[2-(4′-methoxy-phenyl)ethyl]chromone inhibited NF-κB activation in LPS-stimulated RAW 264.7 macrophages with relative luciferase activity values of 0.55 ± 0.09, 0.54 ± 0.03, 0.31 ± 0.05, and 0.38 ± 0.14, respectively, versus that of vehicle control (1.03 ± 0.02). In addition, 5,6-dihydroxy-2-[2-(3′-hydroxy-4′-methoxyphenyl)ethyl]chromone, 7-methoxy-2-(2-phenylethyl)chromone, 7-dimethoxy-2-(2-phenylethyl)chromone, and 6,7-dimethoxy-2-[2-(4′-methoxyphenyl)ethyl]chromone could suppress LPS-induced NO production in RAW 264.7 cells and did not induce cytotoxicity against RAW 264.7 cells after 24-h treatment.

## 1. Introduction

*Aquilaria sinensis* (Lour.) Gilg. (Thymelaeaceae) is an evergreen tree endemic to China. The fragrant resin-infused wood derived from the wounded trees of *Aquilaria* species is called ‘agarwood’ or ‘eaglewood’. Agarwood has been widely used as a traditional sedative, analgesic, and digestive medicine in China [1]. Various benzenoids [2], flavonoids [2,3], 2-(2-phenylethyl)chromones [2,4,5,6,7], sesquiterpenes [8], steroids [2,7], triterpenoids [5], and their derivatives were isolated from this plant in previous studies. Many of these compounds exhibit anti-acetylcholinesterase [8], anti-inflammatory [2,7], antitumor [3], and cytotoxic [6] activities.

**Figure 1:**
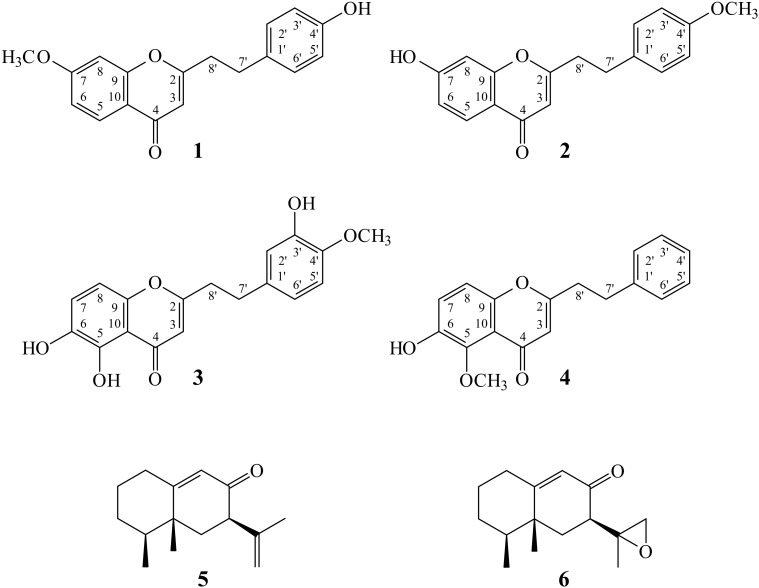

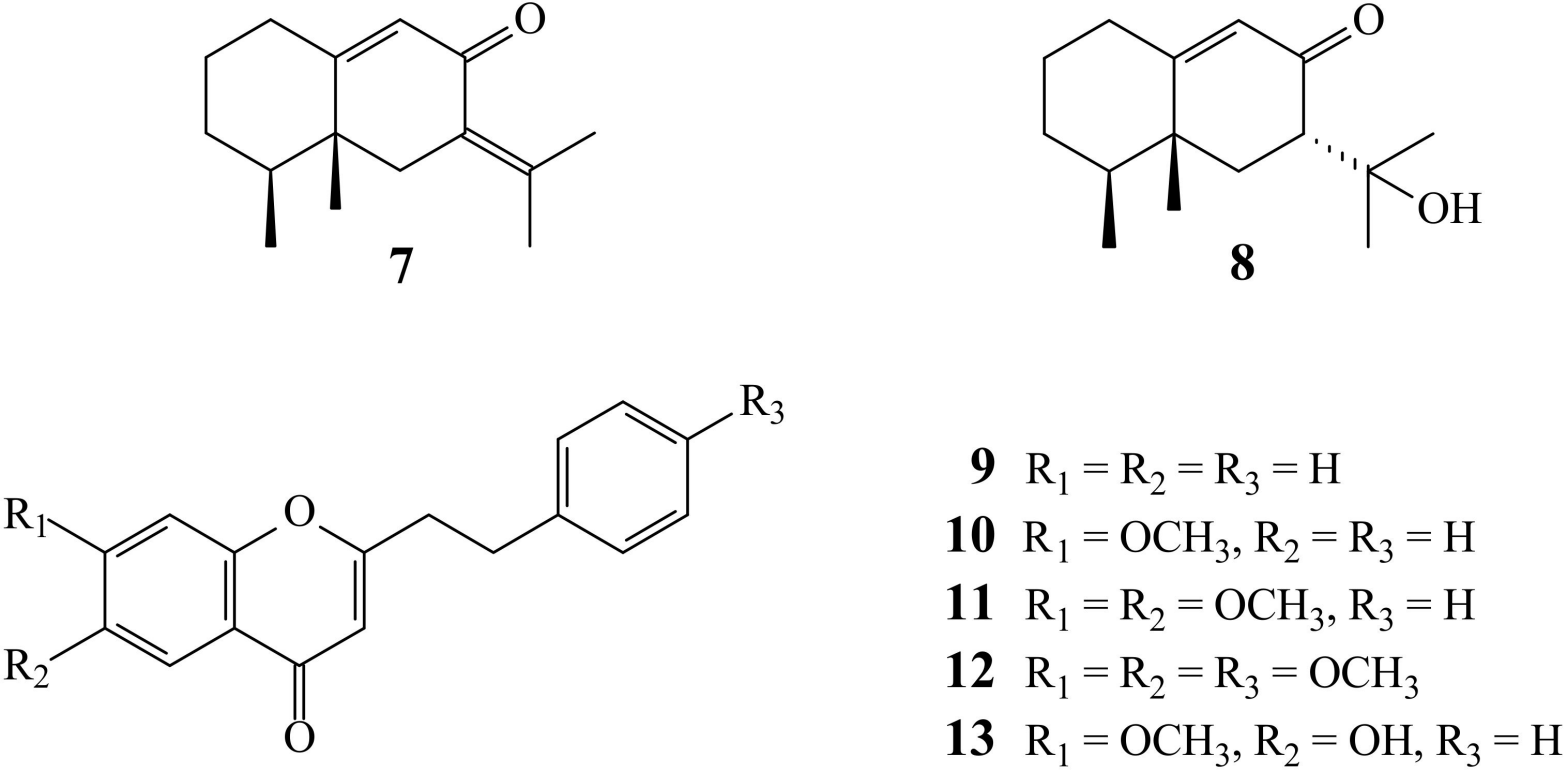
The chemical structures of compounds **1**–**13** isolated from *A. sinensis.*

Aberrant activation of macrophages is related to many inflammatory disorders such as sepsis, neurodegenerative disorders, osteoporosis, cardiovascular and metabolic diseases [9]. Nuclear factor κB (NF-κB) is an important transcription factor when stimulating an inflammatory reaction and as a molecular target for anti-inflammatory drug discovery. Lipopolysaccharide (LPS)-stimulated macrophages have been prevalently applied as an in vitro model system to study inflammation and to identify anti-inflammatory compounds [10,11]. In our studies on the anti-inflammatory constituents of Formosan plants and Chinese herbal medicines, many species have been screened for in vitro inhibitory activity against NF-κB, a key transcriptional activator of pro-inflammatory molecules in LPS-activated macrophages, and *A. sinensis* was found to be an active species. Four new 2-(2-phenylethyl)-4*H*-chromen-4-one derivatives, namely 7-methoxy-2-[2-(4′-hydroxyphenyl)- ethyl]chromone (**1**), 7-hydroxy-2-[2-(4′-methoxyphenyl)ethyl]chromone (**2**), 5,6-dihydroxy-2-[2-(3′-hydroxy-4′-methoxyphenyl)ethyl]chromone (**3**), and 6-hydroxy-5-methoxy-2-(2-phenylethyl)- chromone (**4**), and nine known compounds **5**–**13** have been isolated from the resinous wood of *A. sinensis* and identified. Their structures are depicted in Figure 1. This paper describes the structural elucidation of the compounds numbered **1** through **4** and the inhibitory activities of all isolates on LPS-induced NF-κB activation of macrophages.

## 2. Results and Discussion

### 2.1. Isolation and Structural Elucidation

Chromatographic purification of the EtOAc-soluble fraction of a MeOH extract of resinous wood of *A. sinensis* on a silica gel column and preparative thin-layer chromatography (TLC) afforded four new (**1**–**4**) and nine known compounds (**5**–**13**) (Figure 1).

**Figure 2:**
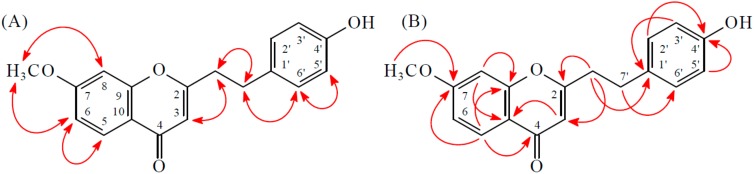
Key NOESY (**A**) and HMBC (**B**) correlations of **1**.

Compound **1** was obtained as brown plates. The molecular formula, C_18_H_16_O_4_, was deduced from a proton adduct ion at *m*/*z* 297.11224 [M + H]^+^ (calcd. 229.11214) in the HR-ESI-MS spectrum (positive-ion mode) and was supported by the ^1^H-, ^13^C-, and DEPT NMR data. IR absorptions for OH (3418 cm^−1^) and γ-pyrone (1634 and 1590 cm^−1^) functions were observed. The ^1^H-NMR spectrum of **1** showed the presence of a methoxy group at δ_H_ 3.91 (3H, s, MeO-7), four methylene protons at δ_H_ 2.86 (2H, t, *J* = 8.0 Hz, H-8′) and 2.98 (2H, t, *J* = 8.0 Hz, H-7′) and a olefinic proton at δ_H_ 6.07 (1H, s, H-3). Additionally a set of typical ABX coupling systems at δ_H_ 6.83 (1H, d, *J* = 2.0 Hz, H-8), 6.95 (1H, dd, *J* = 8.5, 2.0 Hz, H-6) and 8.08 (1H, d, *J* = 8.5 Hz, H-5) and *para*-disubstituted benzene protons at δ_H_ 6.76 (2H, d, *J* = 8.5 Hz, H-3′ and H-5′) and δ_H_ 7.06 (2H, d, *J* = 8.5 Hz, H-2′ and H-6′) were observed. The ^13^C-NMR spectrum of **1** displayed the presence of two methylene groups at δ_C_ 32.0 and 36.3, a trisubstituted double bond at δ_C_ 109.8 and 168.5, a methoxyl group at δ_C_ 56.1, and a carbonyl group at δ_C_ 177.9. Based on the above, **1** was deduced to be a 2-(2-phenylethyl)chromone derivative with a methoxyl and a hydroxyl groups. Two signals at δ_C_ 164.1 and 154.5 arising from *O*-bearing aromatic *C*-atoms were attributable to C-7 and C-4′ respectively, by related HMBC and comparison with a structurally similar compound **10**. The HMBC correlations (Figure 2) observed between OMe-7 (δ_H_ 3.91) and C-7 (δ_C_ 164.1), as well as NOESY correlations (Figure 2) observed between OMe-7 (δ_H_ 3.91) and H-8 (δ_H_ 6.83) and H-6 (δ_H_ 6.95), revealed that the methoxyl group was supposed to be positioned at C-7. Thus, the hydroxyl group should be linked to the last open position at C-4′. Consequently, the structure of **1** was determined to be 7-methoxy-2-[2-(4′-hydroxyphenyl)ethyl]chromone.

**Figure 3:**
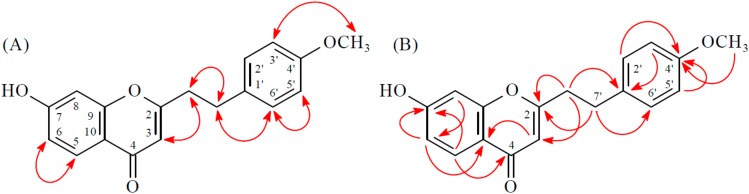
Key NOESY (**A**) and HMBC (**B**) correlations of **2**.

Compound **2** was isolated as a yellowish amorphous powder. The ESI-MS afford the quasi-molecular ion [M + H]^+^ at *m*/*z* 297, implying a molecular formula of C_18_H_17_O_4_, which was confirmed by the HR-ESI-MS (*m*/*z* 297.11227 [M + H]^+^, calcd. 297.11214). The IR spectrum showed the presence of OH (3210 cm^−1^) and γ-pyrone (1635 and 1600 cm^−1^) groups. The ^1^H- and ^13^C-NMR data of **2** were similar to those of 7-methoxy-2-[2-(4′-hydroxyphenyl)ethyl]chromone (**1**), except that the 7-OH and 4′-OMe [δ_H_ 3.79 (3H, s)] groups of **2** replaced the 7-OMe and 4′-OH groups of **1**. This was supported by NOESY correlations between OMe-4′ (δ_H_ 3.79)/H-3′ (δ_H_ 6.83) and OMe-4′ (δ_H_ 3.79)/H-5′ (δ_H_ 6.83) and by HMBC correlations between OMe-4′ (δ_H_ 3.79)/C-4′ (δ_C_ 158.3), H-5 (δ_H_ 8.02)/C-7 (δ_C_ 156.1), and H-6 (δ_H_ 7.09)/C-7 (δ_C_ 156.1). According to the above data, the structure of **2** was elucidated as 7-hydroxy-2-[2-(4′-methoxyphenyl)ethyl]chromone. This was further confirmed by the ^1^H–^1^H-COSY, NOESY (Figure 3), DEPT, HSQC, and HMBC (Figure 3) techniques.

**Figure 4:**
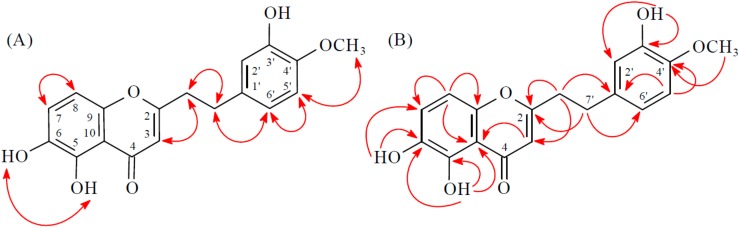
Key NOESY (**A**) and HMBC (**B**) correlations of **3**.

Compound **3** was isolated as a yellowish amorphous powder. Its molecular formula, C_18_H_16_O_6_, was determined on the basis of the positive HRESIMS at *m*/*z* 329.10186 [M + H]^+^ (calcd. 329.10196) and this was supported by the ^1^H, ^13^C, and DEPT NMR data. The IR spectrum showed the presence of OH (3418 cm^−1^) and γ-pyrone (1634 and 1590 cm^−1^) groups. The ^1^H- and ^13^C-NMR data of **3** were similar to those of 3′,6-dihydroxy-4′-methoxy-2-(2-phenylethyl)chromone [7], except that the 5-hydroxy group [δ_H_ 12.50 (1H, s, D_2_O exchangeable)] of **3** replaced H-5 of 3′,6-dihydroxy-4′-methoxy-2-(2-phenylethyl)chromone [7]. This was supported by HMBC correlations between OH-5 (δ_H_ 12.50) and C-5 (δ_C_ 145.3), C-6 (δ_C_ 140.2), and C-10 (δ_C_ 111.0). The full assignment of ^1^H- and ^13^C-NMR resonances was supported by ^1^H–^1^H COSY, DEPT, HSQC, NOESY (Figure 4), and HMBC (Figure 4) spectral analyses. On the basis of the above data, the structure of **3** was elucidated as 5,6-dihydroxy-2-[2-(3′-hydroxy-4′-methoxyphenyl)ethyl]chromone.

**Figure 5:**
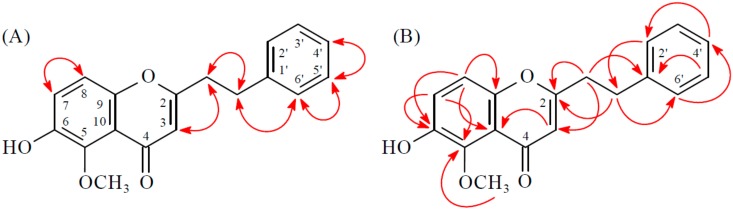
Key NOESY (**A**) and HMBC (**B**) correlations of **4**.

Compound **4** was obtained as a yellowish amorphous powder. The molecular formula C_18_H_16_O_4_ was deduced from a sodium adduct ion at *m*/*z* 319.09395 [M + Na]^+^ (calcd. 319.09408) in the HRESI mass spectrum. The presence of a conjugated carbonyl group was revealed by the band at 1633 cm^−1^ in the IR spectrum, which was confirmed by the resonances at δ_C_ 177.5 in the ^13^C-NMR spectrum. The IR spectrum also revealed a hydroxy absorption at 3340 cm^−1^. Comparison of the ^1^H- and ^13^C-NMR data of **4** with those of 6-hydroxy-2-(2-phenylethyl)chromone (corylifol A) [12] suggested that their structures are closely related, except that the 5-methoxy group [δ_H_ 3.98 (3H, s); δ_C_ 62.8] of **4** replaced H-5 of 6-hydroxy-2-(2-phenylethyl)chromone [12]. This was supported by HMBC correlation between OMe-5 (δ_H_ 3.98) and C-5 (δ_C_ 143.5). The structure elucidation of **4** was supported by ^1^H–^1^H COSY and NOESY (Figure 5) experiments, and ^13^C-NMR assignments were confirmed by DEPT, HSQC, and HMBC (Figure 5) techniques.

### 2.2. Structure Identification of the Known Isolates

The known isolates were readily identified by a comparison of physical and spectroscopic data (UV, IR, ^1^H-NMR, [α]_D_, and MS) with corresponding authentic samples or literature values, and this included four sesquiterpenes, neopetasane (**5**) [13], 7α-*H*-9(10)-ene-11,12-epoxy-8-oxoeremphilane (**6**) [13], dehydrokaranone (**7**) [5], and ligudicin C (**8**) [14], and five 2-(2-phenylethyl)-4*H*-chromen-4-one derivatives, 2-(2-phenylethyl)chromone (**9**) [15], 7-methoxy-2-(2-phenylethyl)chromone (**10**) [16], 6,7-dimethoxy-2-(2-phenylethyl)chromone (**11**) [2], 6,7-dimethoxy-2-[2-(4′-methoxyphenyl)ethyl]chro-mone (**12**) [17], and 6-hydroxy-7-methoxy-2-(2-phenylethyl)chromone (**13**) [18].

### 2.3. Biological Studies

**Figure 6:**
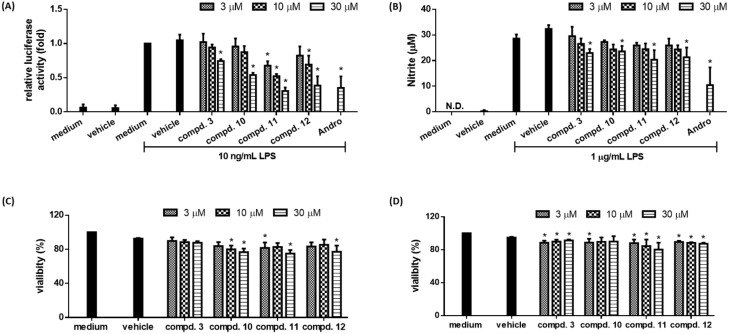
The anti-inflammatory effects of chromone derivatives **3** and **10**–**12** on LPS-stimulated macrophages. (**A**) RAW264.7/Luc-P1 macrophages (1.5 × 10^5^ cells in 24-well plates) were treated with indicated chromone derivatives or 0.1% DMSO for 1 h, followed by LPS (1 μg/mL) treatment for 23 h. The luciferase activity of treated groups was measured; (**B**) RAW 264.7 macrophages (4 × 10^4^ cells in 96-well plates) were treated with indicated compounds or 0.1% DMSO for 1 h, followed by LPS (1 μg/mL) treatment 23 h. Culture supernatants were measured for the production of NO using Griess assays. Andro (andrographolide) is the positive control. Data are expressed as the mean ± SD from three independent experiments. Asterisk (*) indicates significant difference versus LPS-treated vehicle control (*p* < 0.05). The cell viability of RAW 264.7/Luc-P1 cells (1 × 10^4^ cells in 96-well plates) (**C**) and RAW 264.7 cells (1 × 10^4^ cells in 96-well plates) (**D**) incubated with chromone derivatives for 24 h was measured using MTT assay. Data are expressed as the mean ± SD from three independent experiments. Asterisk (*) indicates significant difference versus vehicle control (*p* < 0.05).

**Figure 7:**
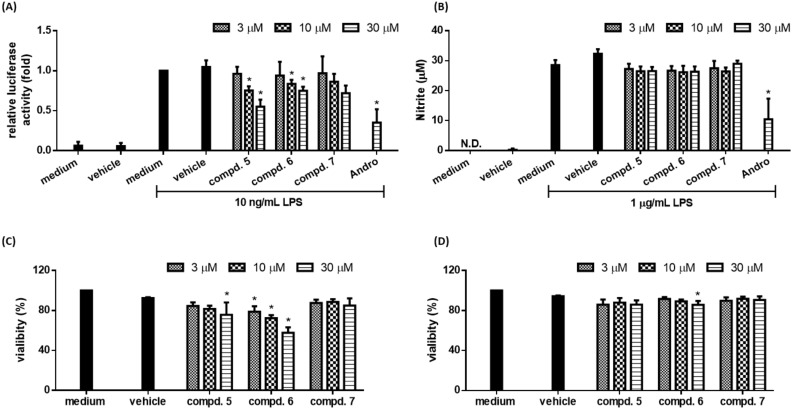
The anti-inflammatory effects of sesquiterpene analogues **5**–**7** on LPS-stimulated macrophages. (**A**) RAW264.7/Luc-P1 macrophages (1.5 × 10^5^ cells in 24-well plates) were treated with indicated sesquiterpenes analogs or 0.1% DMSO for 1 h, followed by LPS (1 μg/mL) treatment for 23 h. The luciferase activity of treated groups was measured; (**B**) RAW 264.7 macrophages (4 × 10^4^ cells in 96-well plates) were treated with indicated compounds or 0.1% DMSO for 1 h, followed by LPS (1 μg/mL) treatment 23 h. Culture supernatants were measured for the production of NO using Griess assays. Andro (andrographolide) is the positive control. Data are expressed as the mean ± SD from three independent experiments. Asterisk (*) indicates significant difference versus LPS-treated vehicle control (*p* < 0.05). The cell viability of RAW 264.7/Luc-P1 cells (1 × 10^4^ cells in 96-well plates) (**C**) and RAW 264.7 cells (1 × 10^4^ cells in 96-well plates) (**D**) incubated with sesquiterpene analogs for 24 h was measured using MTT assay. Data are expressed as the mean ± SD from three independent experiments. Asterisk (*) indicates significant difference versus vehicle control (*p* < 0.05).

LPS-activated macrophage serves as an in vitro model system to study inflammation [19]. A LPS-responsive macrophage cell clone RAW264.7/Luc-P1 was previously established, in which the activity of NF-κB correlates with the expression of reporter gene (*luciferase*) [10]. This RAW 264.7/Luc-P1 cell line has been successfully applied to identify anti-inflammatory compounds [10,19]. Furthermore, LPS-mediated NF-κB activation leads to upregulation of pro-inflammatory molecules, such as NO, in macrophages [20]. Thus, NO production is a hallmark of inflammatory responses. The anti-inflammatory activities of compounds isolated from the resinous wood of *A. sinensis* were evaluated by their abilities to suppress NF-κB activation in RAW 264.7/Luc-P1 cell line, their inhibitory activities are summarized in Table 1. Andrographolide was used as positive control. Based on the results of our bioactivity assays, the following conclusions can be drawn: (a) 5,6-Dihydroxy-2-[2-(3′-hydroxy-4′-methoxyphenyl)ethyl]chromone (**3**), neopetasane (**5**), 7α-*H*-9(10)-ene-11,12-epoxy-8-oxoeremphilane (**6**), dehydrokaranone (**7**), 7-methoxy-2-(2-phenylethyl)chromone (**10**), 6,7-dimethoxy-2-(2-phenylethyl)chromone (**11**), and 6,7-dimethoxy-2-[2-(4′-methoxyphenyl)ethyl]chro-mone (**12**) can significantly inhibit LPS-induced NF-κB activation and did not show cytotoxicity against RAW 264.7/Luc-P1 cells after 24 h treatment (except compound **6** with dose-dependent cytotoxicity in RAW 264.7/Luc-P1 cells) (Figure 6A,C and Figure 7A,C); (b) among the chromone derivatives, 5,6-dihydroxy-2-[2-(3′-hydroxy-4′-methoxyphenyl)ethyl]chromone (**3**), 7-methoxy-2-(2-phenylethyl)chromone (**10**), 7-dimethoxy-2-(2-phenylethyl)chromone (**11**), and 6,7-dimethoxy-2-[2-(4′-methoxyphenyl)ethyl]chromone (**12**) could suppress LPS-induced NO production in RAW264.7 macrophages (Figure 6B) and did not induce cytotoxicity against RAW 264.7 cells after 24-h treatment (Figure 6D); (c) among the sesquiterpene analogues, neopetasan (**5**), 7α-*H*-9(10)-ene-11,12-epoxy-8-oxoeremphilane (**6**), dehydrokaranone (**7**) displayed no inhibitory activity on NO production in RAW264.7 macrophages (Figure 7B) and did not cause significant cytotoxicity (Figure 7D); (d) 6,7-dimethoxy-2-(2-phenylethyl)chromone (**11**) (with a 6-methoxy moiety) exhibited stronger inhibition than its analogue, 6-hydroxy-7-methoxy-2-(2-phenylethyl)chromone (**13**) (with a 6-hydroxy group); (e) 7-methoxy-2-(2-phenylethyl)chromone (**10**) (without the 4′-hydroxy moiety) exhibited more effective inhibition than its analogue, 7-methoxy-2-[2-(4′-hydroxyphenyl)ethyl]chromone (**1**) (with a 4′-hydroxy group); (f) 2-(2-phenylethyl)chromone (**9**) (without any substituents) exhibited no inhibitory activity; (g) among the sesquiterpene analogues **5**, **6**, and **8**, neopetasane (**5**) (with a prop-1-en-2-yl moiety at C-7) exhibited stronger inhibition than the analogues **6** and **8**; (h) 6,7-dimethoxy-2-(2-phenylethyl)chromone (**11**) was the most effective among the isolated compounds, with a relative luciferase activity value of 0.31 ± 0.05, against LPS-induced NF-κB activation in RAW 264.7/Luc-P1 cells.

It is noted that the inhibition on NO production is not always in a tight correlation with NF-κB activation (e.g., compounds **5**, **6**, and **7** in Figure 7B), which may be due to the discrepancy in experimental conditions and the sensitivity of assays used for measuring these two mediators. Alternatively, natural products are commonly known to be multiple-targeted, these compounds may be involved in NF-κB-independent NO regulation.

## 3. Experimental Section

### 3.1. General Procedures

Melting points were determined on a Yanaco micro-melting point apparatus (Yanaco, Tokyo, Japan) and were uncorrected. Optical rotations were measured using a Jasco DIP-370 polarimeter (Japan Spectroscopic Corporation, Tokyo, Japan) in CHCl_3_. Ultraviolet (UV) spectra were obtained on a Jasco UV-240 spectrophotometer. Infrared (IR) spectra (KBr or neat) were recorded on a Perkin Elmer 2000 FT-IR spectrometer (Perkin Elmer Corporation, Norwalk, CT, USA). Nuclear magnetic resonance (NMR) spectra, including nuclear Overhauser effect spectrometry (NOESY), correlation spectroscopy (COSY), heteronuclear single-quantum coherence (HSQC), and heteronuclear multiple-bond correlation (HMBC) experiments, were acquired using a Varian VNMRS-600 or a Varian Inova 500 spectrometer (Varian Inc., Palo Alto, CA, USA) operating at 600 or 500 MHz (^1^H) and 150 or 125 MHz (^13^C), respectively, with chemical shifts given in ppm (δ) using tetramethylsilane (TMS) as an internal standard. Electrospray ionisation (ESI) and high-resolution electrospray ionization (HRESI)-mass spectra were recorded on a VG Platform Electrospray ESI/MS mass spectrometer (Fison, Villeurbanne, France) or a Bruker APEX II (Bruker, Bremen, Germany). Silica gel (70–230, 230–400 mesh, Merck, Darmstadt, Germany) was used for column chromatography (CC). Silica gel 60 F-254 (Merck, Darmstadt, Germany) was used for preparative thin-layer chromatography (PTLC) and thin-layer chromatography (TLC).

### 3.2. Plant Material

The resinous wood of *Aquilaria sinensis* (Lour.) Gilg. (Thymelaeaceae) were collected from Chiayi County, Taiwan, in August 2015 and identified by J.-J. Chen. A voucher specimen (AS-201308) was deposited in the Faculty of Pharmacy, National Yang-Ming University, Taipei, Taiwan.

### 3.3. Extraction and Isolation

The dried resinous wood (600 g) of *A. sinensis* was pulverized and extracted three times with MeOH (15 L each) for 3 days. The extract was concentrated under reduced pressure at 35 °C, and the residue (110 g) was partitioned between EtOAc and H_2_O (1:1) to provide the EtOAc-soluble fraction (fraction A; 65 g). Fraction A (65 g) was purified by CC (3.3 kg of SiO_2_, 70–230 mesh; *n*-hexane/acetone gradient) to afford 9 fractions: A1–A9. Fraction A1 (3.8 g) was subjected to CC (190 g of SiO_2_, 230–400 mesh; *n*-hexane/EtOAc 15:1–0:1, 650 mL-fractions) to give 10 subfractions: A1-1–A1-10. Part (70 mg) of fraction A1-6 was further purified by preparative TLC (SiO_2_; CHCl_3_/EtOAc 60:1) to obtain neopetasane (**5**, 5.2 mg) (R*_f_* = 0.69). Part (85 mg) of fraction A1-10 was further purified by preparative TLC (SiO_2_; CH_2_Cl_2_/EtOAc 15:1) to afford 7α-*H*-9(10)-ene-1,12-epoxy-8-oxoeremphilane (**6**, 5.4 mg) (R*_f_* = 0.67). Fraction A2 (4.8 g) was subjected to CC (240 g of SiO_2_, 230–400 mesh; *n*-hexane/EtOAc 10:1–0:1, 750 mL-fractions) to give 10 subfractions: A2-1–A2-10. Fraction A2-2 (327 mg) was purified by CC (16.5 g of SiO_2_, 230–400 mesh, CHCl_3_/acetone (30:1–0:1), 250 mL-fractions) to give eight sub-fractions: A2-2-1–A2-2-8. Fraction A2-2-2 (35 mg) was further purified by preparative TLC (SiO_2_; CH_2_Cl_2_/EtOAc 30:1) to yield dehydrokaranone (**7**, 4.1 mg) (R*_f_* = 0.60) and 2-(2-phenylethyl)chromone (**9**, 4.6 mg) (R*_f_* = 0.36). Fraction A2-8 (235 mg) was purified by CC (11.8 g of SiO_2_, 230–400 mesh, CHCl_3_/acetone (25:1–0:1), 200 mL-fractions) to give nine subfractions: A2-8-1–A2-8-9. Fraction A2-8-4 (30 mg) was further purified by preparative TLC (SiO_2_; CH_2_Cl_2_/EtOAc 15:1) to afford 7-methoxy-2-(2-phenylethyl)chromone (**10**, 3.4 mg) (R*_f_* = 0.43). Fraction A4 (7.3 g) was subjected to CC (365 g of SiO_2_, 230–400 mesh; CH_2_Cl_2_/acetone 10:1–0:1, 800 mL-fractions) to afford 12 subfractions: A4-1–A4-12. Part (125 mg) of fraction A4-7 was further purified by preparative TLC (SiO_2_; *n*-hexane/EtOAc 1:1) to yield 6,7-dimethoxy-2-(2-phenylethyl)chromone (**11**, 5.4 mg) (R*_f_* = 0.48) and ligudicin C (**8**, 5.7 mg) (R*_f_* = 0.35). Fraction A5 (7.7 g) was subjected to CC (385 g of SiO_2_, 230–400 mesh; *n*-hexane/EtOAc 5:1–0:1, 700 mL-fractions) to give 11 subfractions: A5-1–A5-11. Part (110 mg) of fraction A5-6 was further purified by preparative TLC (SiO_2_; CHCl_3_/acetone 20:1) to obtain 6,7-dimethoxy-2-[2-(4′-methoxyphenyl)ethyl]chromone (**12**, 8.2 mg) (R*_f_* = 0.62). Fraction A7 (7.2 g) was subjected to CC (360 g of SiO_2_, 230–400 mesh; *n*-hexane/acetone 4:1–0:1, 700 mL-fractions) to give 10 subfractions: A7-1–A7-10. Fraction A7-7 (515 mg) was purified by CC (25.5 g of SiO_2_, 230–400 mesh, CH_2_Cl_2_/EtOAc (10:1–0:1), 500 mL-fractions) to give eight subfractions: A7-7-1–A7-7-8. Fraction A7-7-2 (33 mg) was further purified by preparative TLC (SiO_2_; *n*-hexane/acetone 3:2) to obtain 7-methoxy-2-[2-(4′-hydroxy-phenyl)ethyl]chromone (**1**, 2.5 mg) (R*_f_* = 0.53), 7-hydroxy-2-[2-(4′-methoxyphenyl)ethyl]chromone (**2**, 2.8 mg) (R*_f_* = 0.47), and 6-hydroxy-7-methoxy-2-(2-phenylethyl)chromone (**13**, 3.9 mg) (R*_f_* = 0.43). Part (95 mg) of fraction A7-9 was further purified by preparative TLC (SiO_2_; CH_2_Cl_2_/MeOH 20:1) to yield 5,6-dihydroxy-2-[2-(3′-hydroxy-4′-methoxyphenyl)ethyl]chromone (**3**, 3.7 mg) (R*_f_* = 0.55). Fraction A8 (8.6 g) was subjected to CC (435 g of SiO_2_, 230–400 mesh; hexane/acetone 2:1–0:1, 900 mL-fractions) to afford 13 subfractions: A8-1–A8-13. Fraction A8-2 (620 mg) was purified by CC (31 g of SiO_2_, 230–400 mesh, *n*-hexane/EtOAc (3:1–0:1), 400 mL-fractions) to give seven subfractions: A8-2-1–A8-2-7. Part (82 mg) of fraction A8-2-6 was further purified by preparative TLC (SiO_2_; *n*-hexane/EtOAc 1:1) to obtain 6-hydroxy-5-methoxy-2-(2-phenylethyl)chromone (**4**, 4.4 mg) (R*_f_* = 0.48).

*7-Methoxy-2-[2-(4′-hydroxyphenyl)ethyl]chromone* (**1**): brown plates; m.p. 155–157 °C (CH_2_Cl_2_-MeOH); UV (MeOH): λ_max_ (log ε) = 219 (4.64), 247 (4.41), 282 (4.31), 302 (4.21) nm; IR (KBr): υ_max_ = 3418 (OH), 1634, 1590 (γ-pyrone) cm^−1^; ^1^H-NMR (CDCl_3_, 500 MHz): see Table 2; ^13^C-NMR (CDCl_3_, 125 MHz): see Table 3; ESI-MS: *m*/*z* = 297 [M + H]^+^; HR-ESI-MS: *m*/*z* = 297.11224 [M + H]^+^ (calcd. for C_18_H_17_O_6_, 297.11214).

*7-Hydroxy-2-[2-(4′-methoxyphenyl)ethyl]chromone* (**2**): yellowish amorphous powder; UV (MeOH): λ_max_ (log ε) = 222 (4.69), 251 (4.42), 303 (4.16) nm; IR (KBr): υ_max_ = 3210 (OH), 1635, 1600 (γ-pyrone) cm^−1^; ^1^H-NMR (CDCl_3_, 500 MHz): see Table 2; ^13^C-NMR (CDCl_3_, 125 MHz): see Table 3; ESI-MS: *m*/*z* = 297 [M + Na]^+^; HR-ESI-MS: *m*/*z* = 297.11227 [M + H]^+^ (calcd. for C_18_H_17_O_6_: 297.11214).

*5,6-Dihydroxy-2-[2-(3′-hydroxy-4′-methoxyphenyl)ethyl]chromone* (**3**): yellowish amorphous powder; UV (MeOH): λ_max_ (log ε) = 204 (4.83), 235 (4.52), 358 (3.68) nm; IR (KBr): υ_max_ = 3418 (OH), 1634, 1590 (γ-pyrone) cm^−1^; ^1^H-NMR (CDCl_3_, 500 MHz): see Table 2; ^13^C-NMR (CDCl_3_, 125 MHz): see Table 3; ESI-MS: *m*/*z* = 329 [M + Na]^+^; HR-ESI-MS: *m*/*z* = 329.10186 [M + H]^+^ (calcd. for C_18_H_17_O_6_: 329.10196).

*6-Hydroxy-5-methoxy-2-(2-phenylethyl)chromone* (**4**): yellowish amorphous powder; UV (MeOH): λ_max_ (log ε) = 203 (4.40), 238 (4.37), 334 (3.71) nm; IR (KBr): υ_max_ = 3340 (OH), 1633, 1609 (γ-pyrone) cm^−1^; ^1^H-NMR (CDCl_3_, 600 MHz): see Table 2; ^13^C-NMR (CDCl_3_, 150 MHz): see Table 3; ESI-MS: *m*/*z* = 319 [M + Na]^+^; HR-ESI-MS: *m*/*z* = 319.09395 [M + Na]^+^ (calcd. for C_18_H_16_O_4_Na: 319.09408).

### 3.4. Biological Assay

The inhibitory effects of the isolated compounds on LPS-induced NF-κB activation in RAW 264.7/Luc-P1 macrophage were evaluated by measuring the luminescence resulted from luciferase activity in a concentration-dependent manner. The purity of the tested compounds was >98% as identified by NMR and MS.

#### 3.4.1. Cells and Culture Medium

RAW 264.7/Luc-P1 cell was an LPS-responsive cell line with an integrated reporter gene (pELAM1-Luc). The cell line was cultured under conditions as described previously [10]. The RAW 264.7/Luc-P1 cells were regularly cultured at 37 °C in 5% CO_2_ incubator in DMEM supplemented with 10% heat-inactivated BCS, 100 U/mL penicillin, 100 μg/mL streptomycin, 2 μM L-glutamine, and 1 mM sodium pyruvate.

#### 3.4.2. Luciferase Reporter Assay

The RAW 264.7/Luc-P1 cells (1.5 × 10^5^ cells in 24-well plates) were treated with isolated compounds, vehicle (0.1% DMSO) or the positive control (30 μM andrographolide) for 1 h and then LPS (10 ng/mL) for 23 h, collected, and analyzed using luciferase assays (Promega, Madison, WI, USA). Cell lysates (20 μL) were then mixed with 100 μL luciferin right before luminescence detection [11]. The luminescence was measured with an Infinite^®^ 200 PRO (Tecan Group Ltd., Männedorf, Switzerland).

#### 3.4.3. Cytotoxicity Assay

The MTT assay was used according to a previously described method [21]. RAW 264.7 cells or RAW 264.7/Luc-P1 cells (1 × 10^4^ cells) were seeded into 96-well plates and treated with compounds (**3**, **5**–**7**, and **10**–**12**) and 0.1% DMSO for 24 h.

#### 3.4.4. Determination of Nitric Oxide (NO) Production

RAW 264.7 cells (4 × 10^4^ cells) were seeded into 96-well plates and treated with compounds (**3**, **5**–**7**, and **10**–**12**) and 0.1% DMSO for 1 h, then incubated with LPS (1 μg/mL) for 23 h. The 100 μL of cell culture medium was incubated with 100 μL of Griess reagent at room temperature for 10 min. The absorbance was measured at 550 nm against a calibration curve with sodium nitrite (NaNO_2_) standards.

#### 3.4.5. Statistical Analysis

Data are shown as mean ± SD of three independent experiments. Statistical analysis was performed using ANOVA followed by post hoc Dunnett’s test to compare between-group differences. Differences were considered as statistically significant when *p* < 0.05.

## 4. Conclusions

Thirteen compounds, including four new 2-(2-phenylethyl)-4*H*-chromen-4-one derivatives **1**–**4** were isolated from the resinous wood of *A. sinensis*. The structures of these compounds were established on the basis of spectroscopic data. The activity assays revealed that compounds **3**, **5**–**7**, and **10**–**12** inhibited LPS-stimulated NF-κB activation with relative luciferase activity values of 0.74 ± 0.03, 0.55 ± 0.09, 0.75 ± 0.05, 0.72 ± 0.10, 0.54 ± 0.03, 0.31 ± 0.05, and 0.38 ± 0.14, respectively. Moreover, chromone derivatives could suppress LPS-induced NO production without causing significant cytotoxicity. Our study suggests that agarwood and its isolates (especially compound **11**) are worthy of further biomedical investigation and could be developed as potential candidates for the treatment or prevention of various inflammatory diseases. The structure-and-activity relationship (SAR) of these isolated compounds in term of anti-inflammatory activity certainly merits further investigation.

## Figures and Tables

**Table 1:** The effects of compounds **1**–**13** from the resinous wood of *A. sinensis* on NF-κB activation in RAW 264.7/Luc-P1 cells.

Compounds ^a^	Relative Luciferase Activity
	Mean ± SD ^c^
1	0.89 ± 0.05
2	0.93 ± 0.06
3	0.74 ± 0.03 *
4	0.92 ± 0.01
5	0.55 ± 0.09 *
6	0.75 ± 0.05 *
7	0.72 ± 0.16 *
8	0.98 ± 0.07
9	1.26 ± 0.40
10	0.54 ± 0.03 *
11	0.31 ± 0.05 *
12	0.38 ± 0.14 *
13	1.09 ± 0.21
LPS-treated vehicle control ^b^	1.03 ± 0.02
Andrographolide ^d^	0.35 ± 0.17 *

^a^ Compounds **1**–**13**: 30 μM. ^b^ Vehicle control: 0.1% DMSO. ^c^ Data are expressed as the mean ± SD from three independent experiments. * indicates significant difference versus LPS (1 μg/mL)-treated vehicle control (*p* < 0.05). ^d^ Andrographolide (30 μM) is the positive control.

**Table 2:** ^1^H-NMR data for compounds **1**–**4** (δ in ppm, *J* in Hz).

Position	1 ^a^	2 ^a^	3 ^a^	4 ^b^
3	6.07 s	6.16 s	6.02 s	6.06 s
5	8.08 d (8.5)	8.02 d (9.0)	–	–
6	6.95 dd (8.5, 2.0)	7.09 dd (9.0, 2.5)	–	–
7	–	–	7.28 d (9.0)	7.30 d (9.3)
8	6.83 d (2.0)	6.84 d (2.5)	6.86 d (9.0)	7.14 d (9.3)
2′	7.06 d (8.5)	7.13 d (8.5)	6.78 d (2.0)	7.20 d (7.8)
3′	6.76 d (8.5)	6.83 d (8.5)	–	7.30 t (7.8)
4′	–	–	–	7.22 t (7.8)
5′	6.76 d (8.5)	6.83 d (8.5)	6.76 d (8.0)	7.30 t (7.8)
6′	7.06 d (8.5)	7.13 d (8.5)	6.64 dd (8.0, 2.0)	7.20 d (7.8)
7′	2.98 t (8.0)	3.05 t (8.0)	2.96 t (7.0)	3.04 t (7.8)
8′	2.86 t (8.0)	2.96 t (8.0)	2.88 t (7.0)	2.88 t (7.8)
OH-5	–	–	12.50 s	–
OH-6	–	–	5.43 s	–
OH-3′	–	–	5.58 s	–
OMe-5	–	–	–	3.98 s
OMe-7	3.91 s	–	–	–
OMe-4′	–	3.79	3.87 s	–

^a^ Measured in CDCl_3_ at 500 MHz. ^b^ Measured in CDCl_3_ at 600 MHz.

**Table 3:** ^13^C-NMR data for compounds **1**–**4** (δ in ppm).

Position	1 ^a^	2 ^a^	3 ^a^	4 ^b^
2	168.5	168.1	170.4	167.0
3	109.8	110.3	108.2	110.5
4	177.9	177.1	183.2	177.5
5	127.2	125.2	145.3	143.5
6	116.5	113.8	140.2	146.1
7	164.1	156.1	121.6	120.7
8	100.1	103.9	110.7	114.1
9	158.1	156.6	144.0	151.4
10	115.4	118.4	111.0	117.9
1′	131.5	131.5	129.5	139.8
2′	129.4	129.2	115.9	128.3
3′	115.6	114.1	144.6	128.7
4′	154.5	158.3	145.9	126.6
5′	115.6	114.1	112.0	128.7
6′	129.4	129.2	123.9	128.3
7′	32.0	31.9	30.3	32.9
8′	36.3	36.1	34.7	35.6
OMe-5	–	–	–	62.8
OMe-7	56.1	–	–	–
OMe-4′	–	55.3	56.3	–

^a^ Measured in CDCl_3_ at 125 MHz. ^b^ Measured in CDCl_3_ at 150 MHz.

## Supplementary Materials

Supplementary materials are available online, Figures S1–S8: MS, 1D, and 2D-NMR spectra for 7-methoxy-2-[2-(4′-hydroxyphenyl)ethyl]chromone (**1**), Figures S9–S16: MS, 1D, and 2D-NMR spectra for 7-hydroxy-2-[2-(4′-methoxyphenyl)ethyl]chromone (**2**), Figures S17–S24: MS, 1D, and 2D-NMR spectra for 5,6-dihydroxy-2-[2-(3′-hydroxy-4′-methoxyphenyl)ethyl]chromone (**3**), Figures S25–S32: MS, 1D, and 2D-NMR spectra for 6-hydroxy-5-methoxy-2-(2-phenylethyl)chromone (**4**).
