# Supplementary material for: 2-(2-Phenylethyl)-4H-chromen-4-one Derivatives from the Resinous Wood of Aquilaria sinensis with Anti-Inflammatory Effects in LPS-Induced Macrophages
Source: Molecules. 2018 Jan 30;23(2):289. doi: 10.3390/molecules23020289 (PMC6017433; doi:10.3390/molecules23020289)
Supplement: Supplementary file 1 [file molecules-23-00289-s001.pdf]

Supplementary Materials

2-(2-Phenylethyl)-4*H*-chromen-4-one Derivatives from the Resinous Wood of *Aquilaria sinensis* with Anti-inflammatory Effect in LPS-induced Macrophages

Sin-Ling Wang [1], Yun-Chen Tsai [2], Shu-Ling Fu [2,†], Mei-Ing Chung [1,†,*] and Jih-Jung Chen [3,4,*]

1 School of Pharmacy, College of Pharmacy, Kaohsiung Medical University, Kaohsiung 807, Taiwan; s8332805@yahoo.com.tw (S.-L.W.)
2 Institute of Traditional Medicine, National Yang-Ming University, Taipei 112, Taiwan; tyc202006@gmail.com (Y.-C.T.); slfu@ym.edu.tw (S.-L.F.)
3 Faculty of Pharmacy, School of Pharmaceutical Sciences, National Yang-Ming University, Taipei 112, Taiwan
4 Department of Medical Research, China Medical University Hospital, China Medical University, Taichung 404, Taiwan

* Correspondence: chenjj@ym.edu.tw (J.-J.C.); Tel.: +886-2-2826-7195; meinch@kmu.edu.tw (M.-I.C.); Tel.: +886-7-312-1101 (ext. 2672)

† Authors have contributed equally in this manuscript.

Contents

Figure S1. ESI-MS spectrum of **1** S2
Figure S2. HR-ESI-MS spectrum of **1** S3
Figure S3. ^{1}H-NMR spectrum ($CDCl_3$, 500 MHz) of **1** S3
Figure S4. ^{13}C-NMR spectrum ($CDCl_3$, 125 MHz) of **1** S4
Figure S5. ^{1}H-^{1}H COSY spectrum of **1** S4
Figure S6. NOESY spectrum of **1** S5
Figure S7. HSQC spectrum of **1** S5
Figure S8. HMBC spectrum of **1** S6
Figure S9. ESI-MS spectrum of **2** S6
Figure S10. HR-ESI-MS spectrum of **2** S7
Figure S11. ^{1}H-NMR spectrum ($CDCl_3$, 500 MHz) of **2** S7
Figure S12. ^{13}C-NMR spectrum ($CDCl_3$, 125 MHz) of **2** S8
Figure S13. ^{1}H-^{1}H COSY spectrum of **2** S8
Figure S14. NOESY spectrum of **2** S9
Figure S15. HSQC spectrum of **2** S9
Figure S16. HMBC spectrum of **2** S10
Figure S17. ESI-MS spectrum of **3** S10
Figure S18. HR-ESI-MS spectrum of **3** S11
Figure S19. ^{1}H-NMR spectrum ($CDCl_3$, 500 MHz) of **3** S11
Figure S20. ^{13}C-NMR spectrum (CDCl3, 125 MHz) of **3** S12
Figure S21. ^{1}H-^{1}H COSY spectrum of **3** S12
Figure S22. NOESY spectrum of **3** S13
Figure S23. HSQC spectrum of **3** S13
Figure S24. HMBC spectrum of **3** S14
Figure S25. ESI-MS spectrum of **4** S14
Figure S26. HR-ESIMS spectrum of **4** S15
Figure S27. ^{1}H-NMR spectrum ($CDCl_3$, 600 MHz) of **4** S15

Figure S28. ^{13}C-NMR spectrum ($CDCl_3$, 150 MHz) of **4** S16
Figure S29. ^{1}H-^{1}H COSY spectrum of **4** S16
Figure S30. NOESY spectrum of **4** S17
Figure S31. HSQC spectrum of **4** S17
Figure S32. HMBC spectrum of **4** S18
Figure S33. The data of cell viability after andrographolide treatment..S18

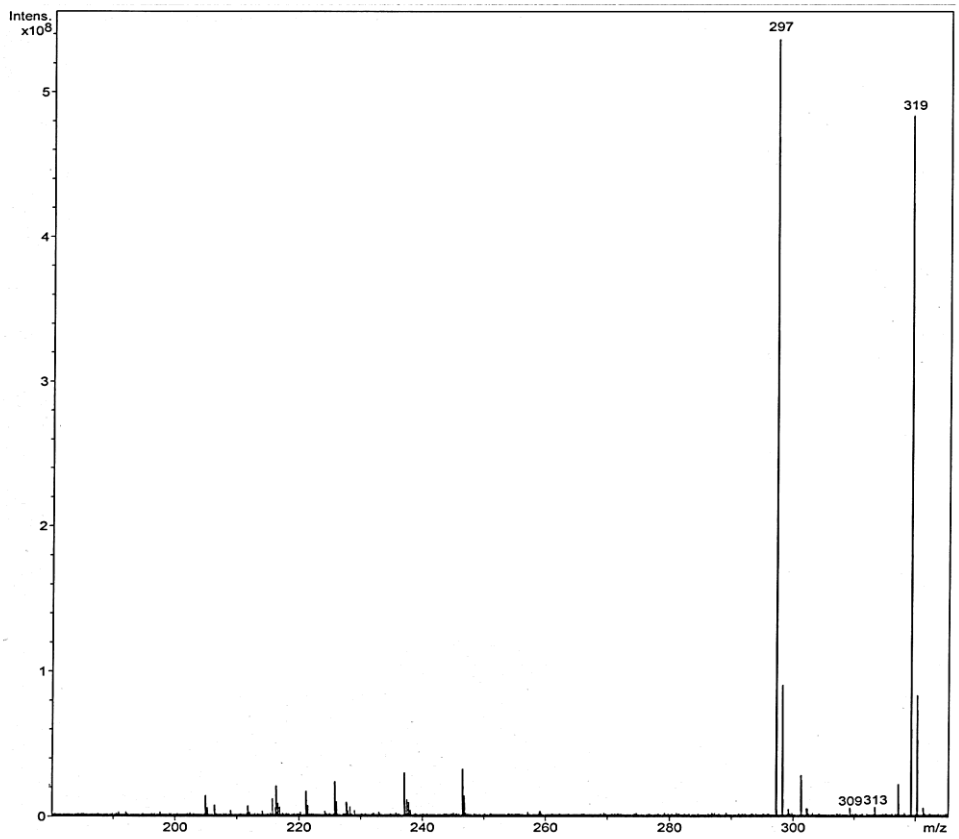

Figure S1. ESI-MS spectrum of **1**.

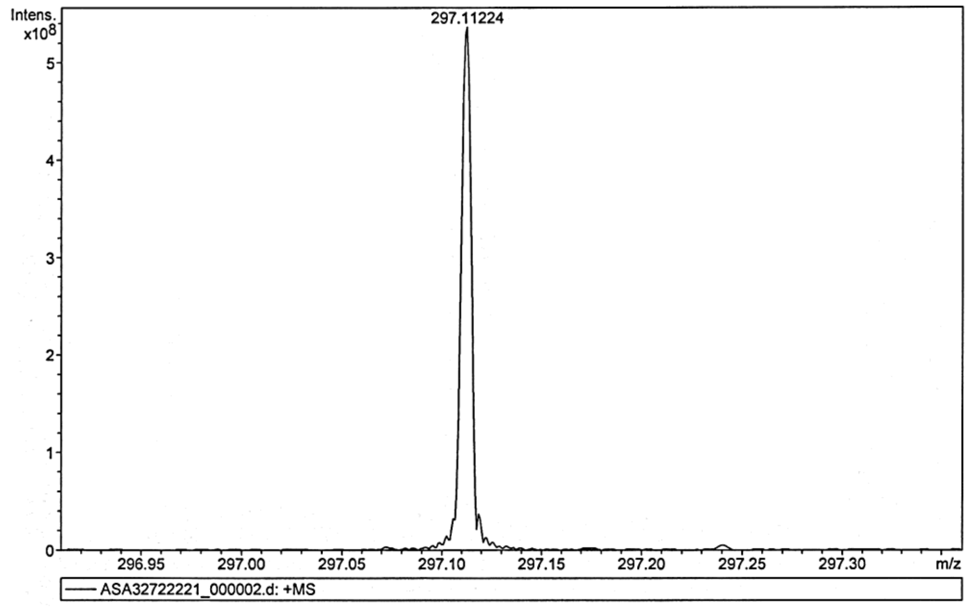

Meas. m/z	#	Formula	Score	m/z	err [mDa]	err [ppm]	mSigma	rdb	e⁻ Conf	N-Rule
297.11224	1	C 18 H 17 O 4	100.00	297.11214	-0.11	-0.36	17.7	10.5	even	ok

Figure S2. HR-ESI-MS spectrum of **1**.

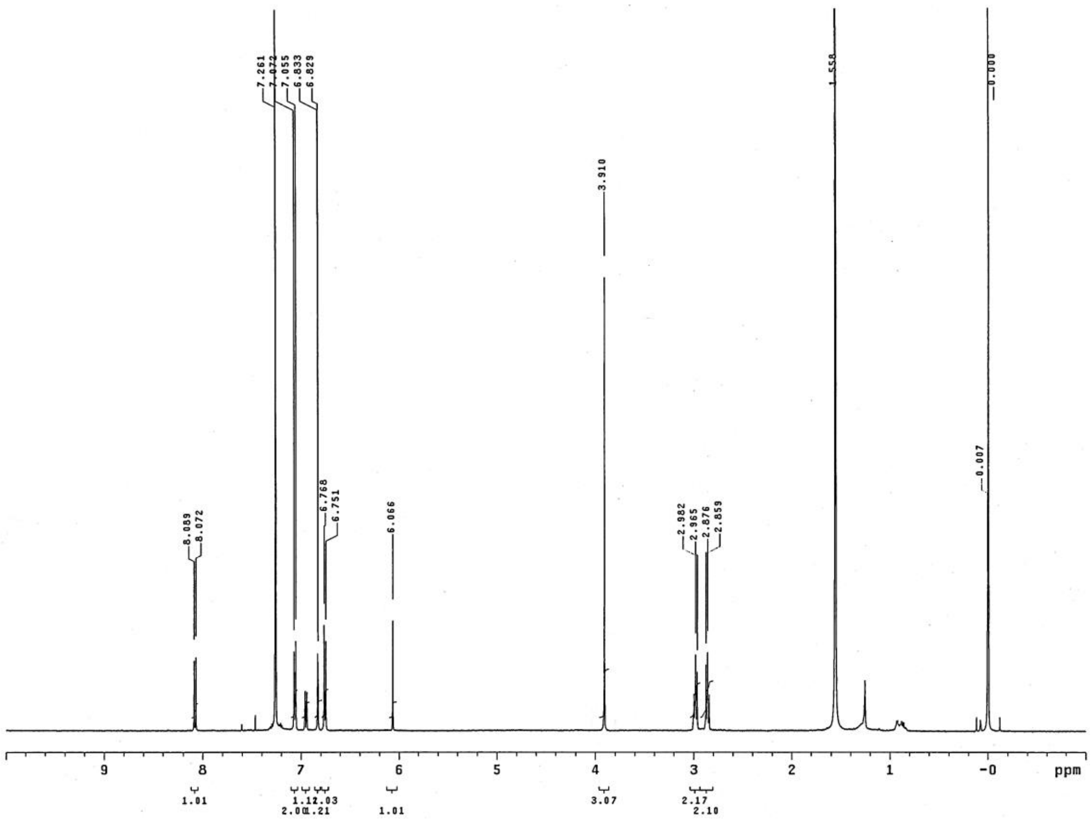

Figure S3. ^{1}H-NMR spectrum ($CDCl_3$, 500 MHz) of **1**.

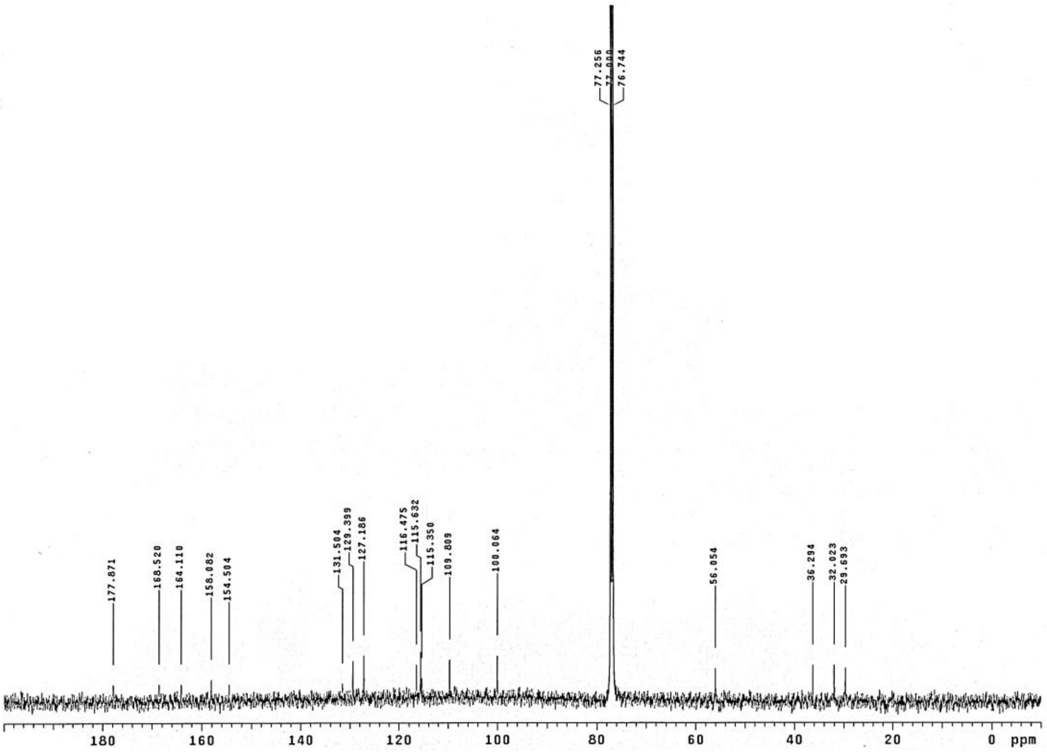

Figure S4. ^{13}C-NMR spectrum ($CDCl_3$, 125 MHz) of **1**.

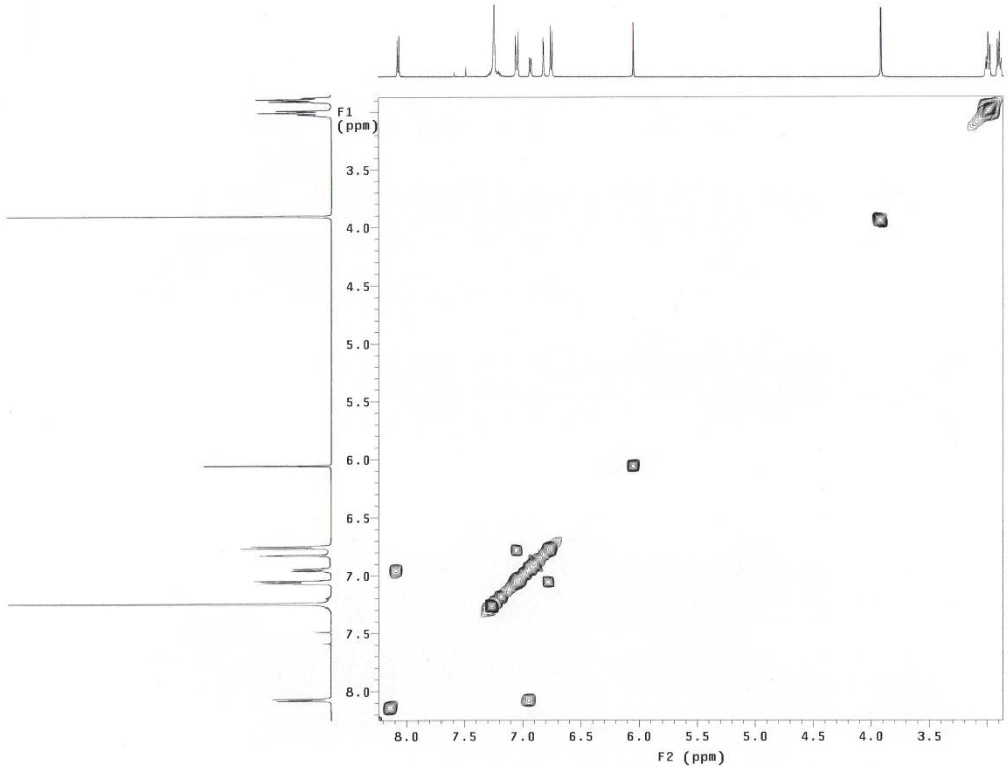

Figure S5. ^{1}H–^{1}H COSY spectrum of **1**.

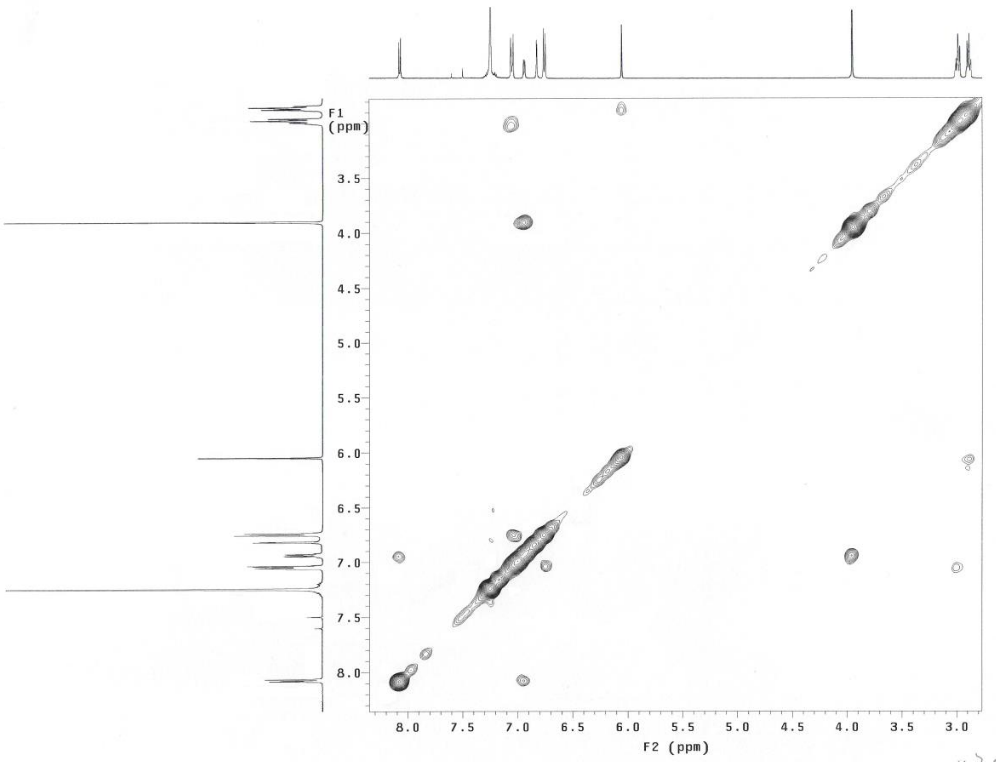

Figure S6. NOESY spectrum of **1.**

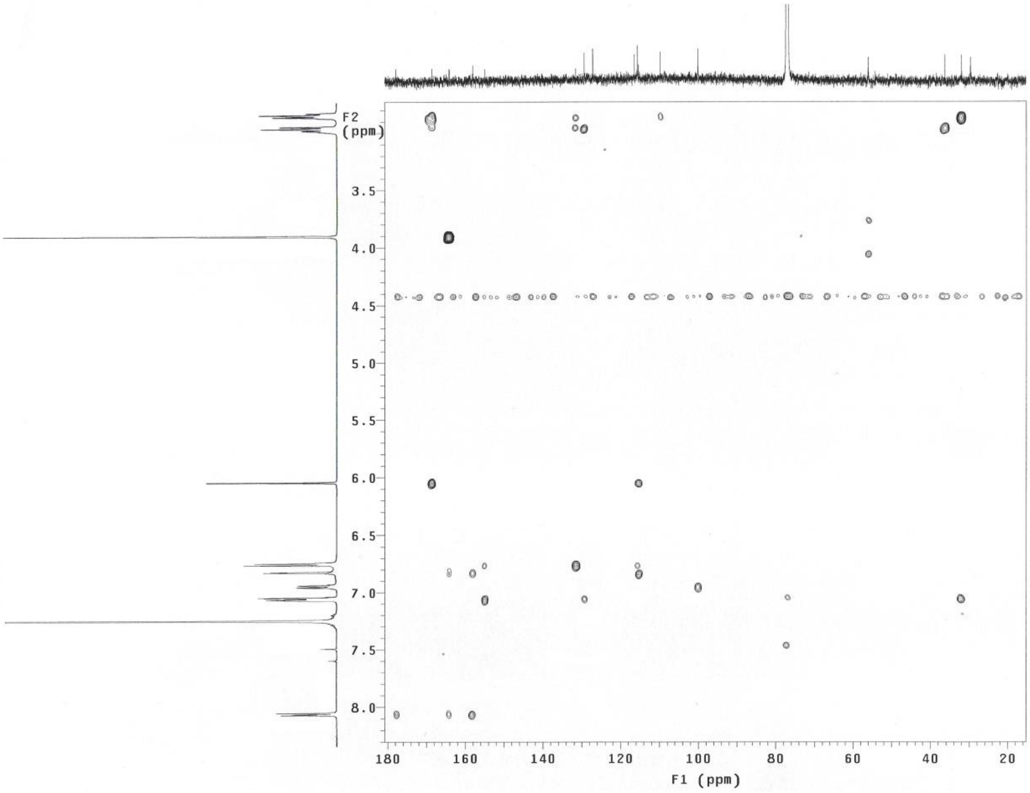

Figure S7. HMBC spectrum of **1**.

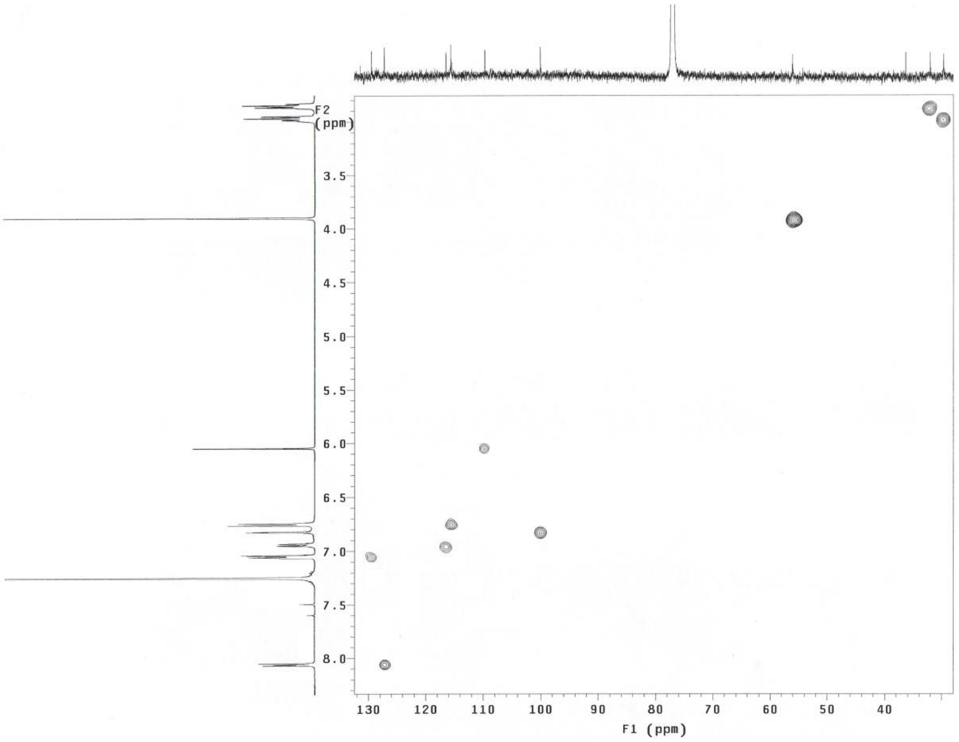

Figure S8. HSQC spectrum of **1**.

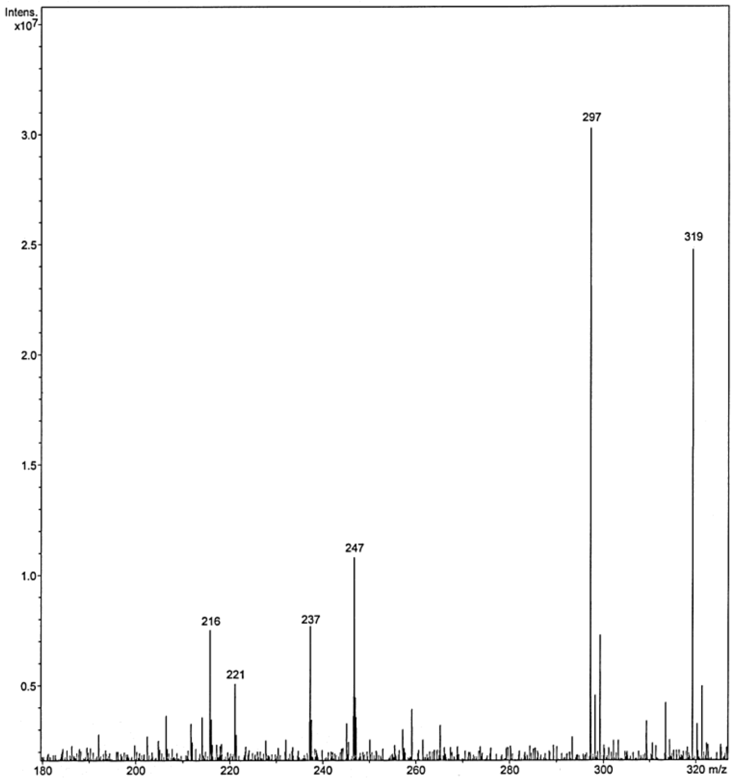

Figure S9. ESI-MS spectrum of **2**.

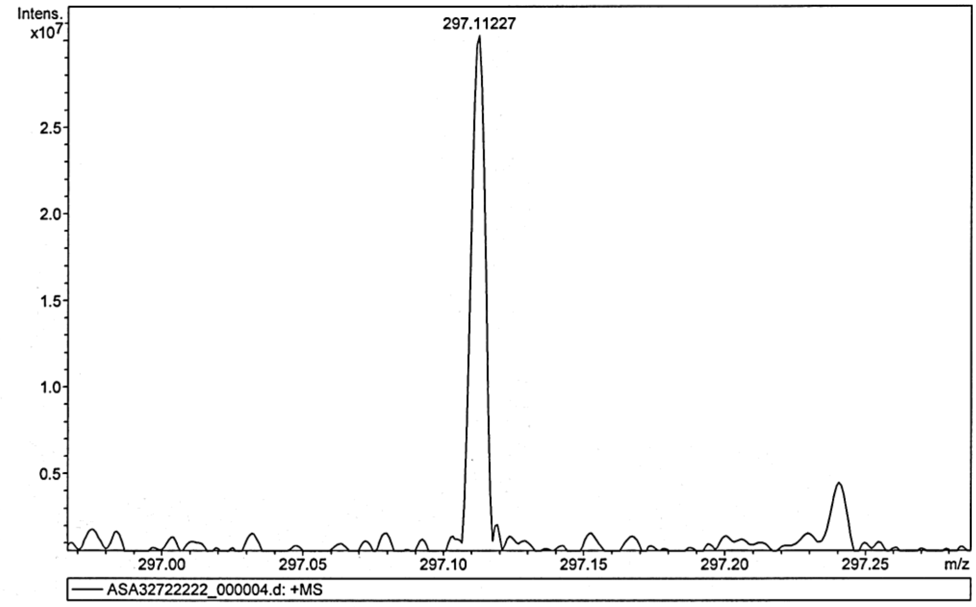

Meas. m/z	#	Formula	Score	m/z	err [mDa]	err [ppm]	mSigma	rdb	e^- Conf	N-Rule
297.11227	1	C 18 H 17 O 4	100.00	297.11214	-0.14	-0.46	15.5	10.5	even	ok

Figure S10. HR-ESI-MS spectrum of **2**.

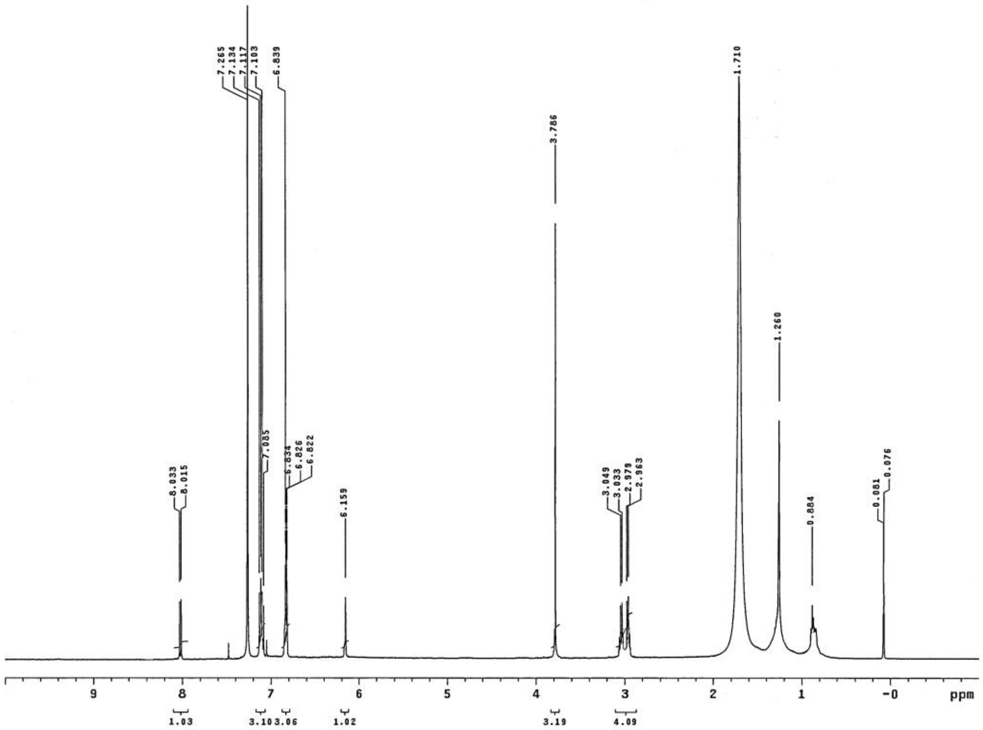

Figure S11. ^{1}H-NMR spectrum ($CDCl_3$, 500 MHz) of **2**.

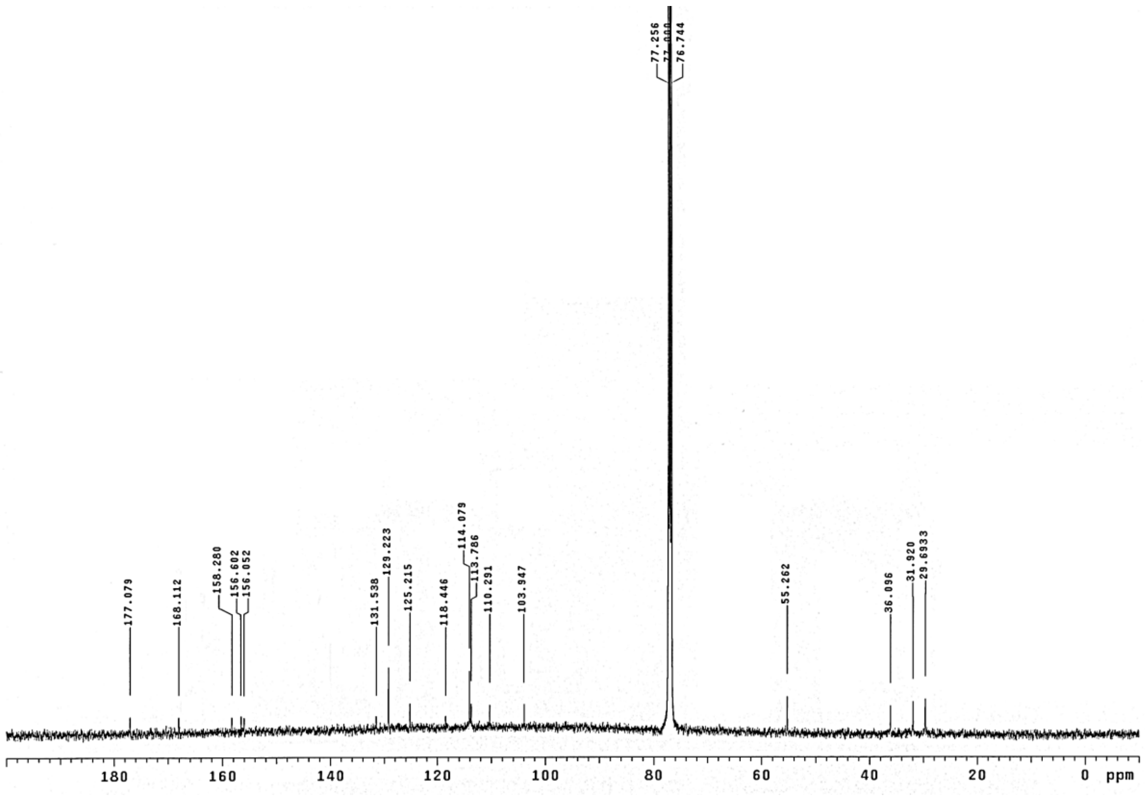

Figure S12. ^{13}C-NMR spectrum ($CDCl_3$, 125 MHz) of **2**.

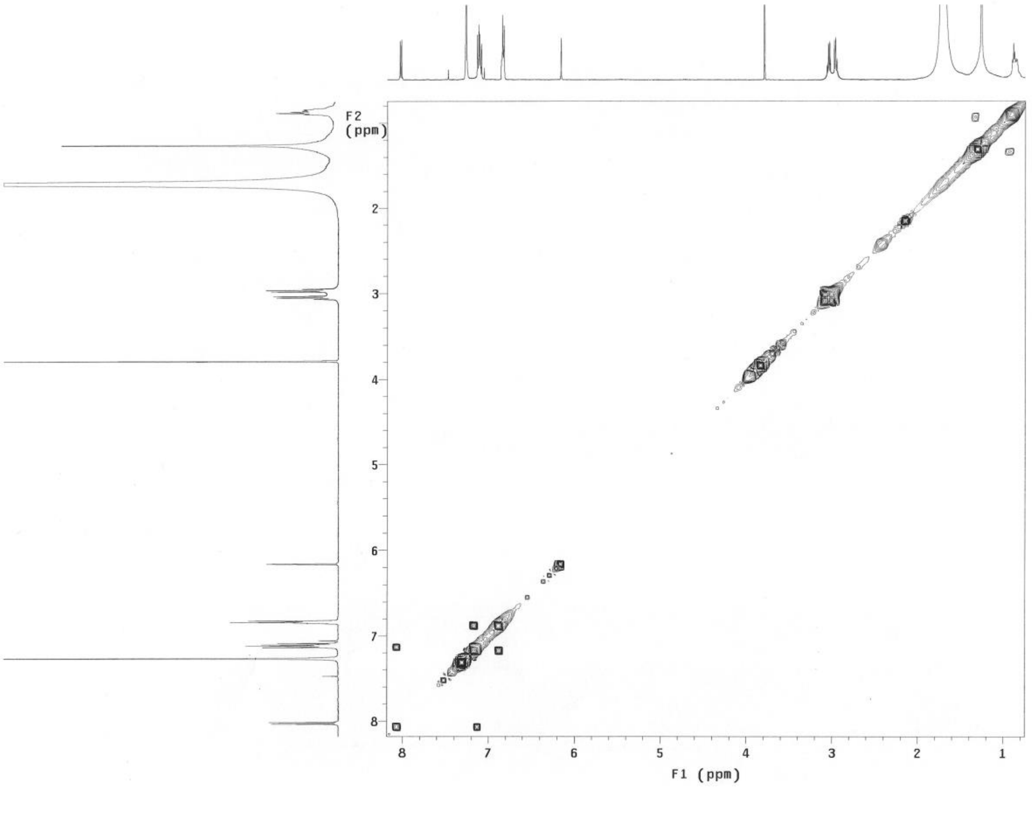

Figure S13. ^1H–^1H COSY spectrum of **2**.

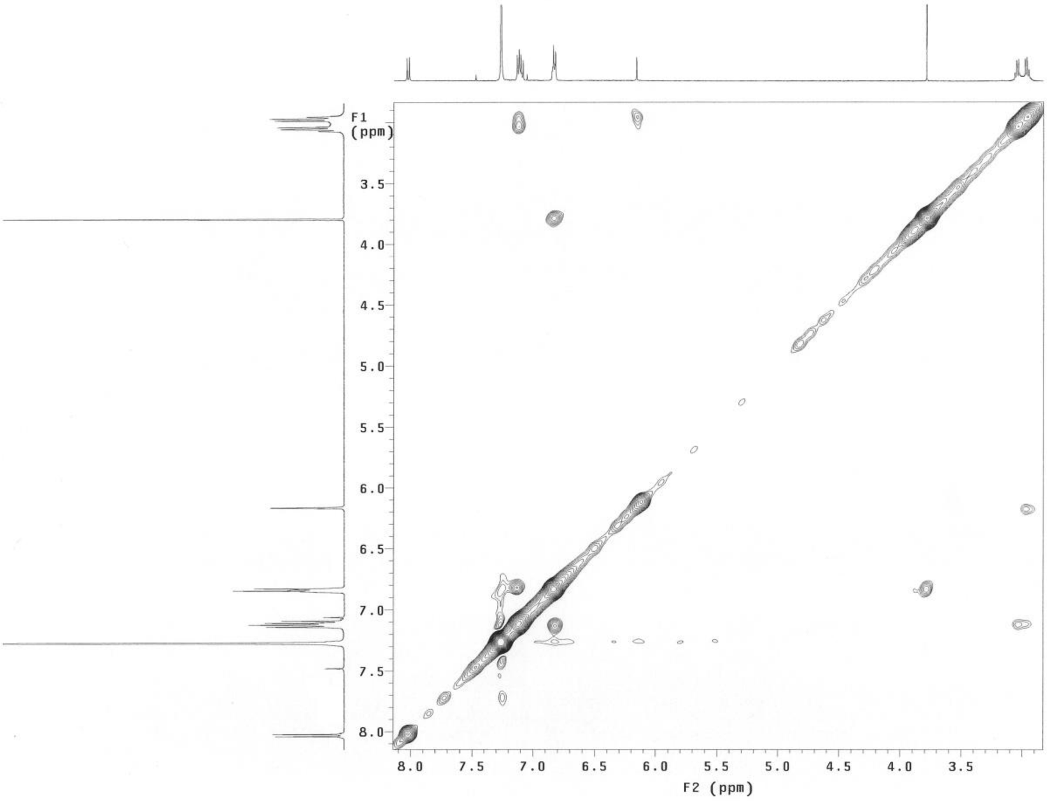

Figure S14. NOESY spectrum of **2**.

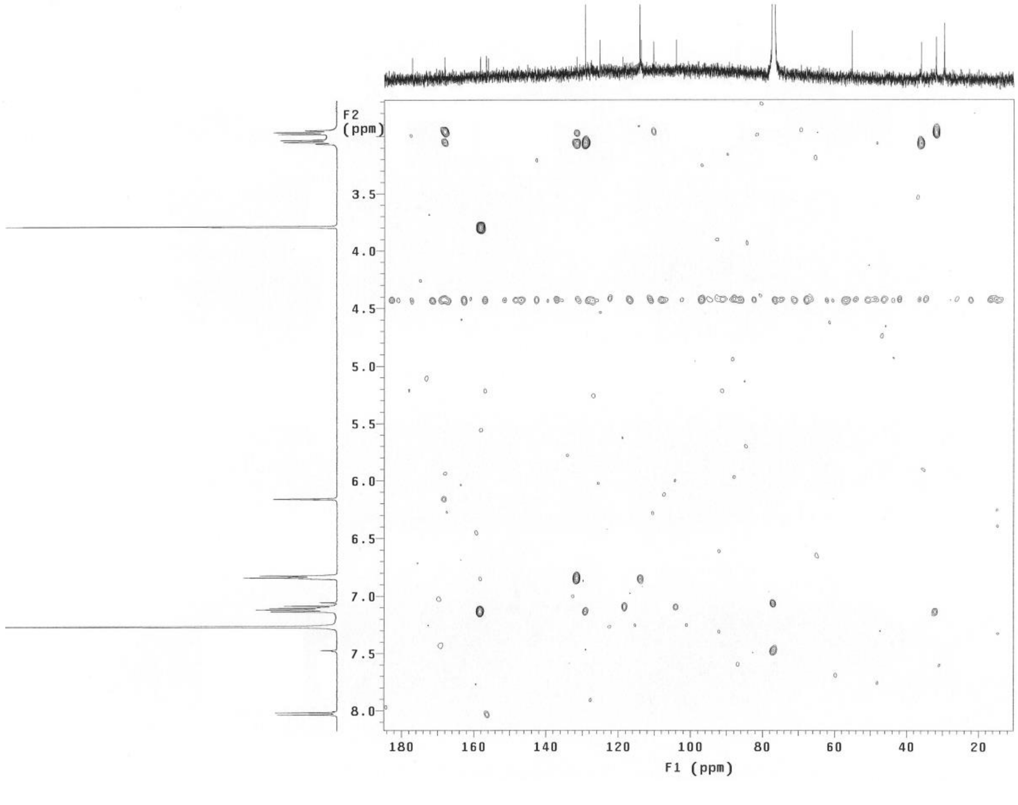

Figure S15. HMBC spectrum of **2**.

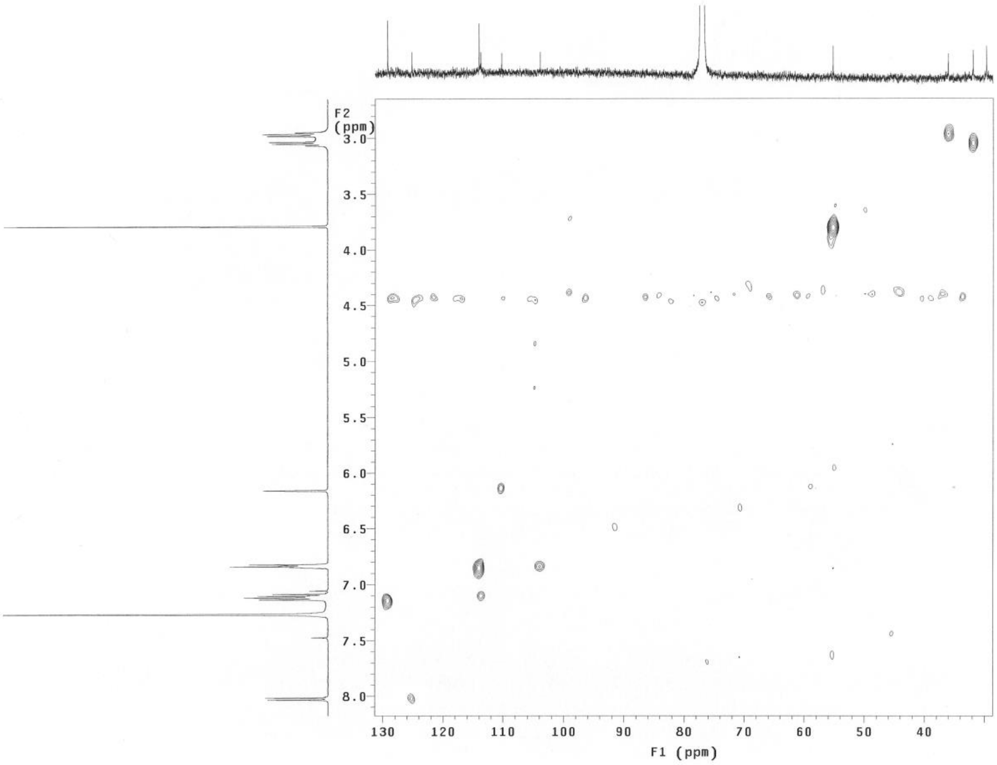

Figure S16. HSQC spectrum of **2**.

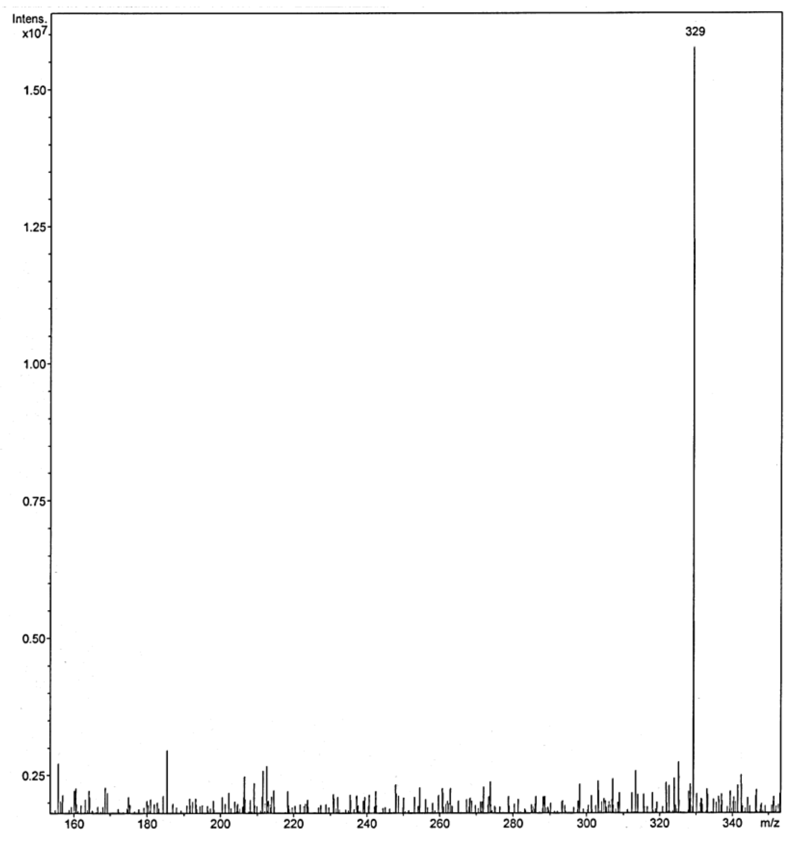

Figure S17. ESI-MS spectrum of **3**.

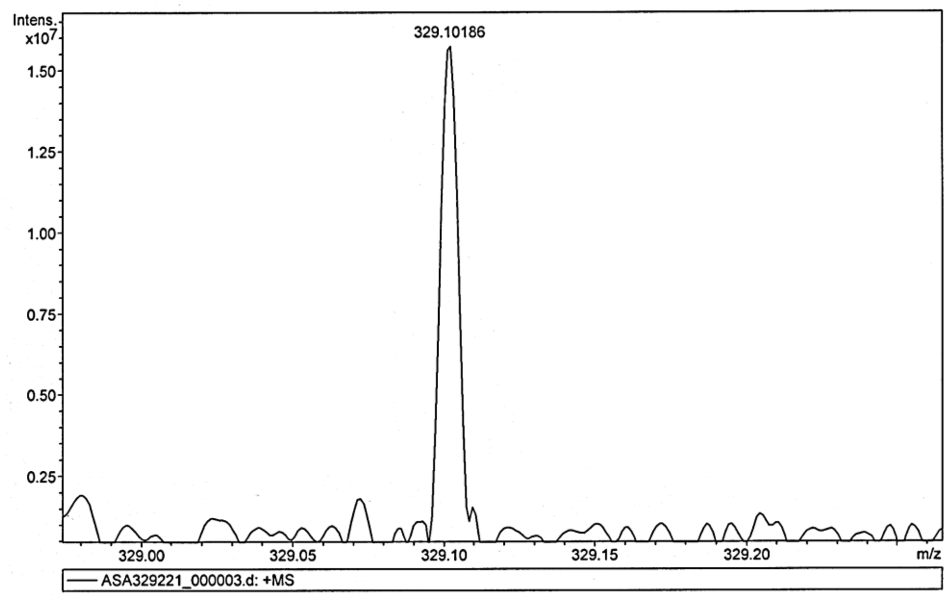

Meas. m/z	#	Formula	Score	m/z	err [mDa]	err [ppm]	mSigma	rdb	e⁻ Conf	N-Rule
329.10186	1	C 18 H 17 O 6	100.00	329.10196	0.11	0.32	37.9	10.5	even	ok

Figure S18. HR-ESI-MS spectrum of **3**.

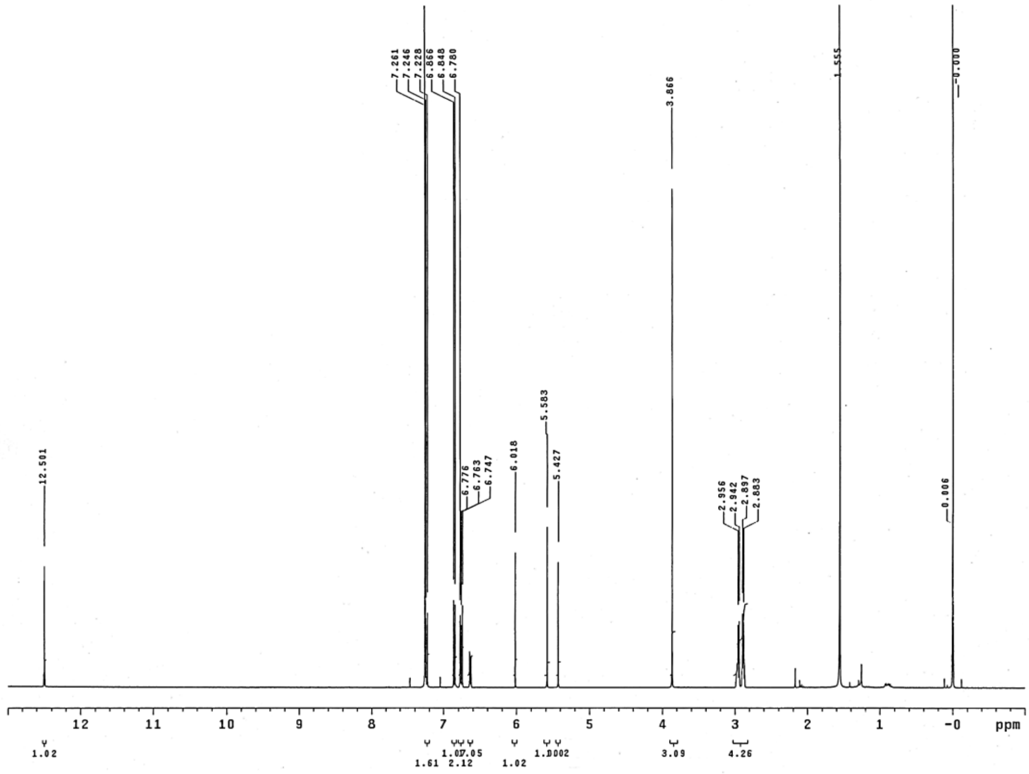

Figure S19. ^{1}H-NMR spectrum ($CDCl_3$, 500 MHz) of **3**.

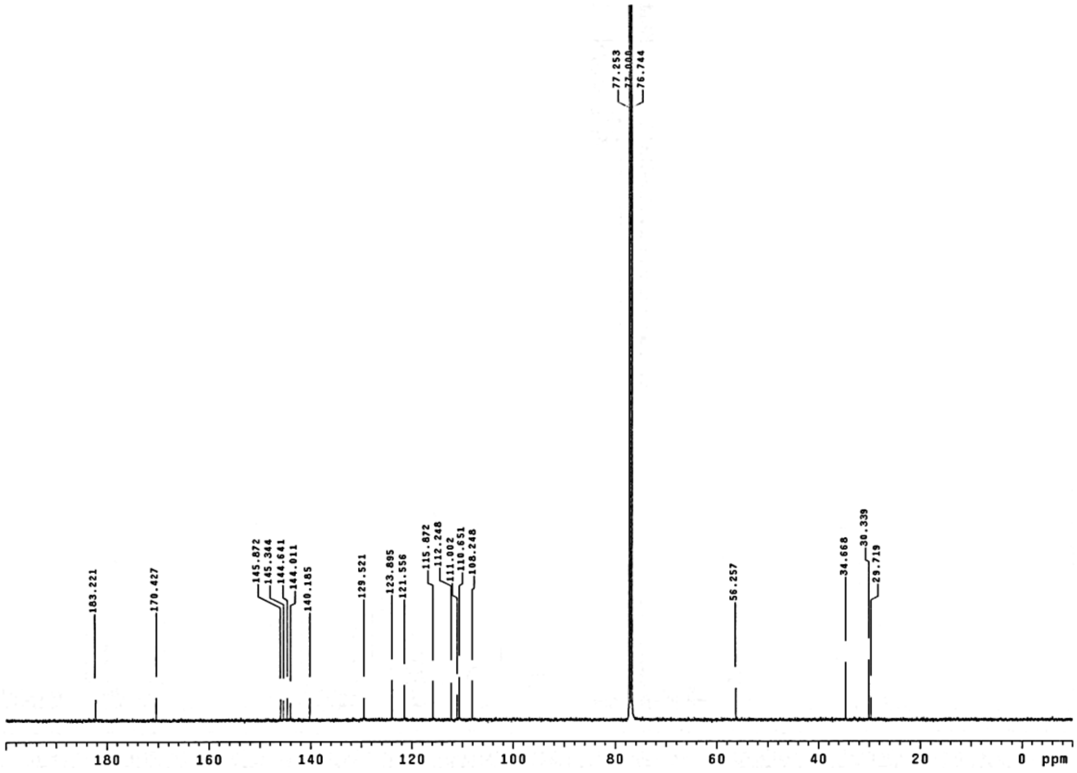

Figure S20. ^{13}C-NMR spectrum of **3** ($CDCl_3$, 125 MHz

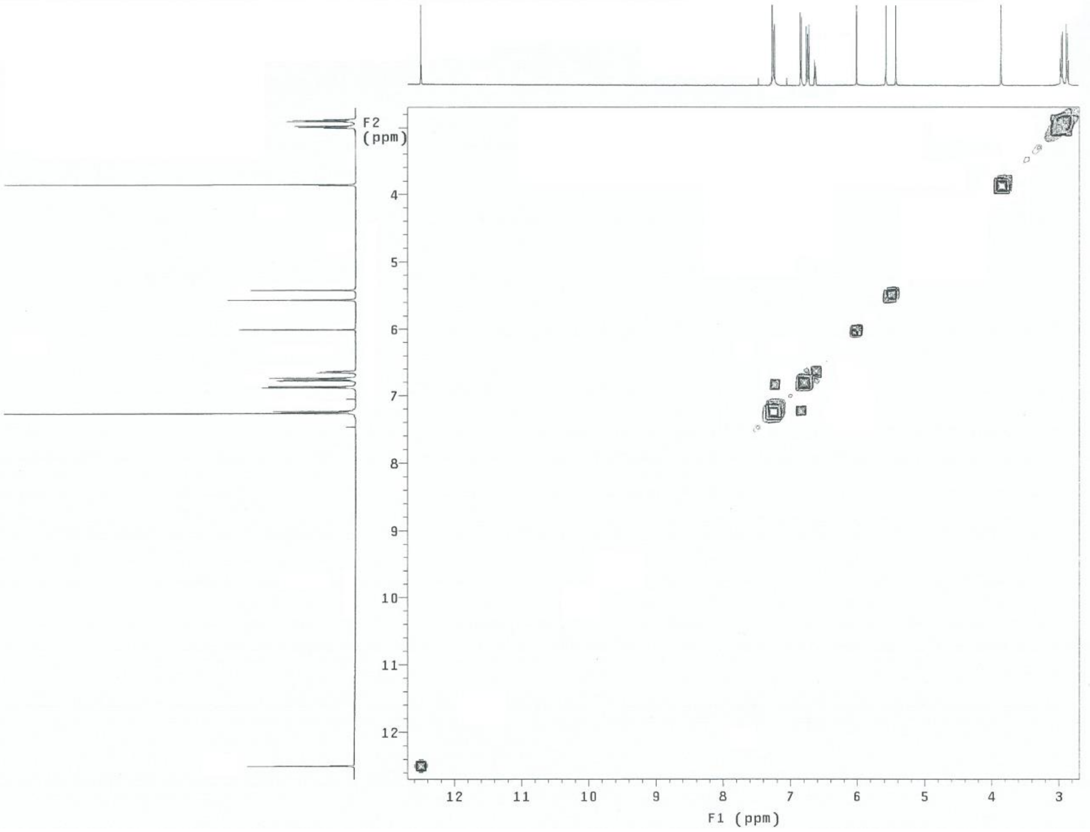

Figure S21. ^{1}H–^{1}H COSY spectrum of **3**.

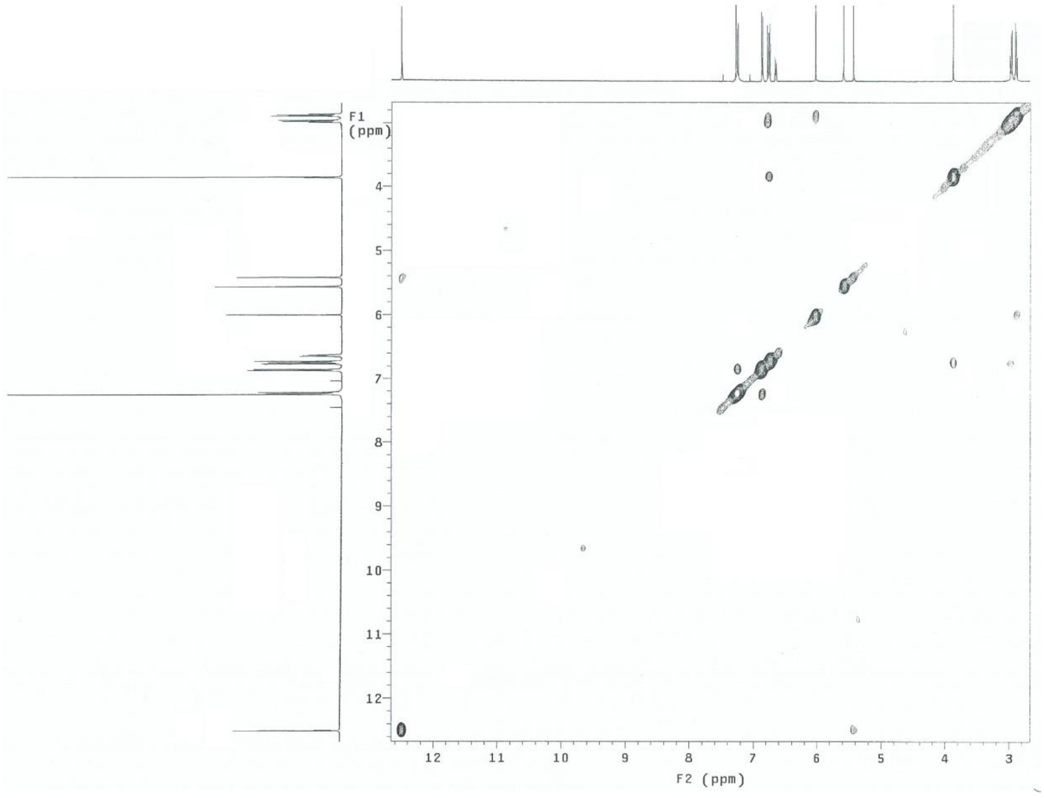

Figure S22. NOESY spectrum of **3**.

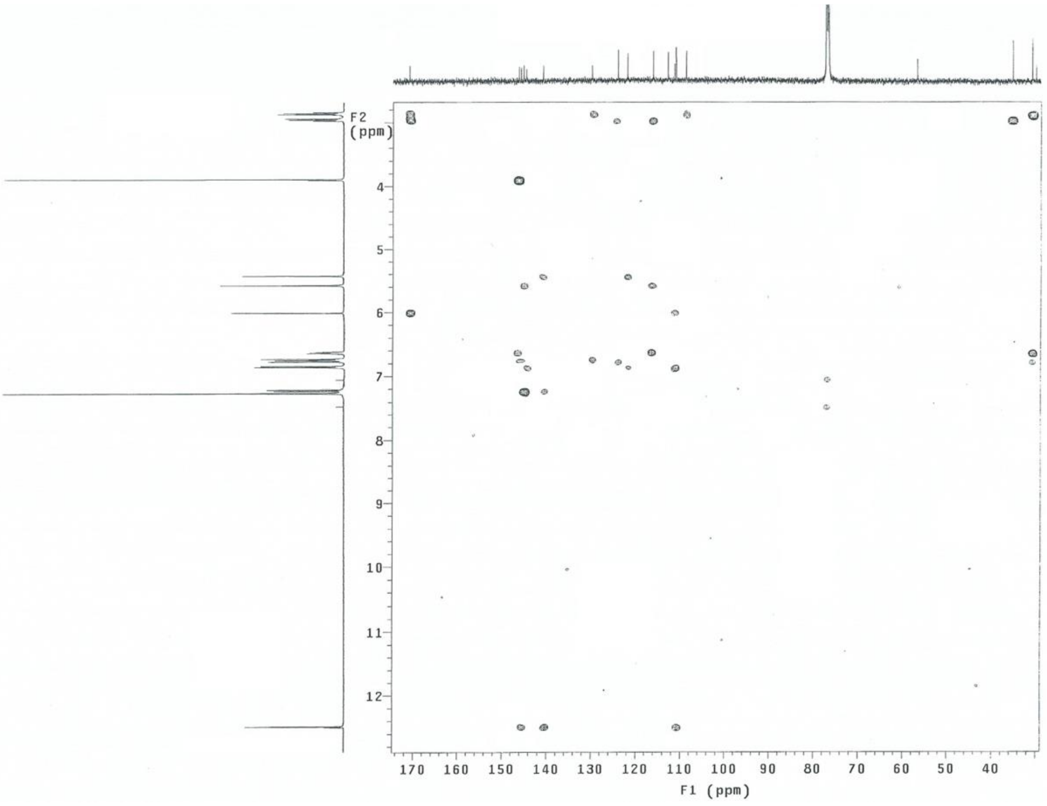

Figure S23. HMBC spectrum of **3**.

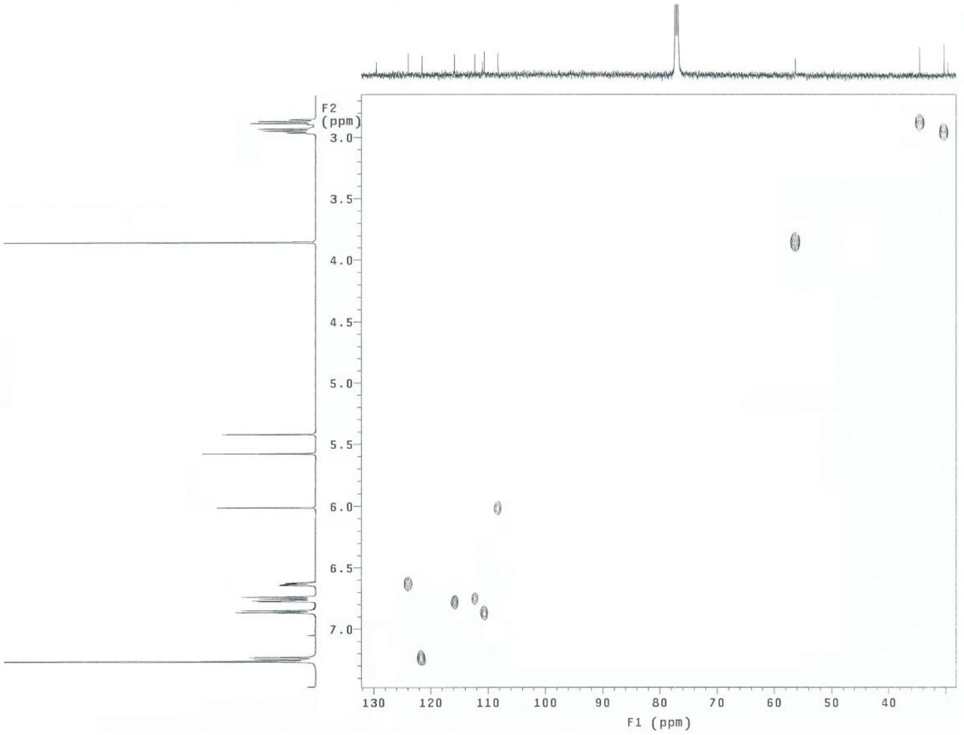

Figure S24. HSQC spectrum of **3**.

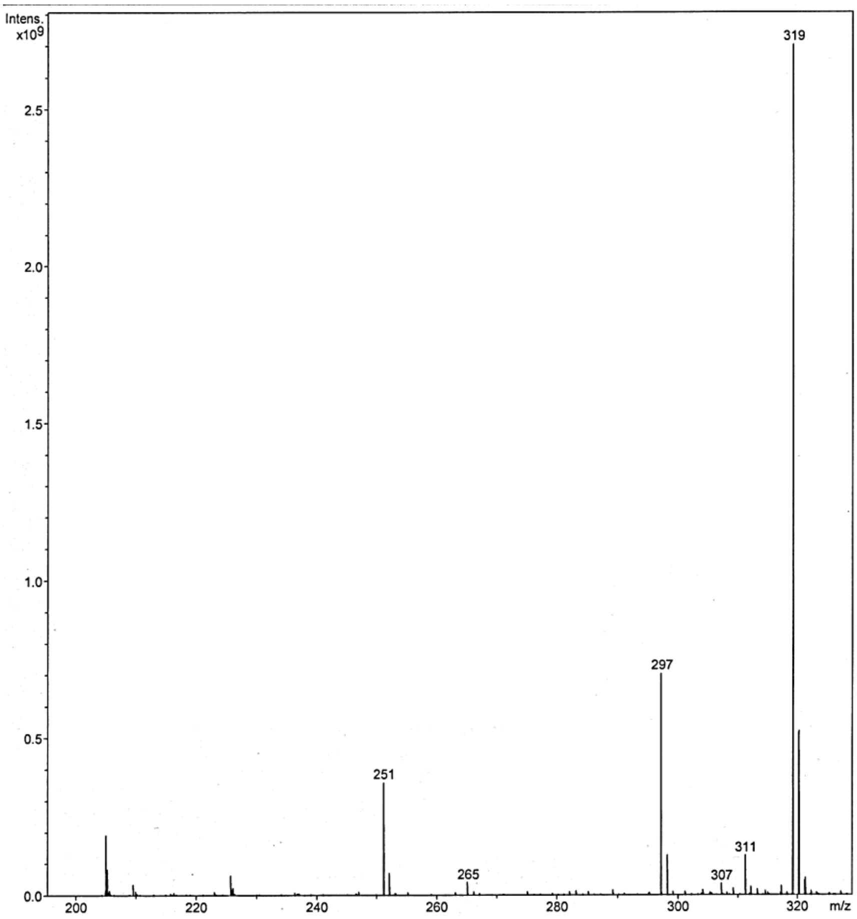

Figure S25. ESI-MS spectrum of **4**

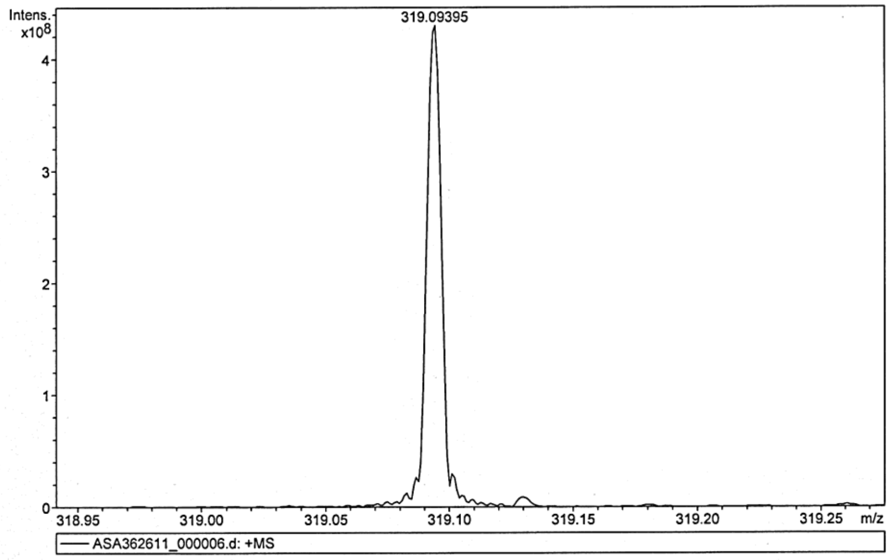

Meas. m/z	#	Formula	Score	m/z	err [mDa]	err [ppm]	mSigma	rdb	e⁻ Conf	N-Rule
319.09395	1	C 18 H 16 Na O 4	100.00	319.09408	0.13	0.42	5.7	10.5	even	ok

Figure S26. HR-ESI-MS spectrum of **4**.

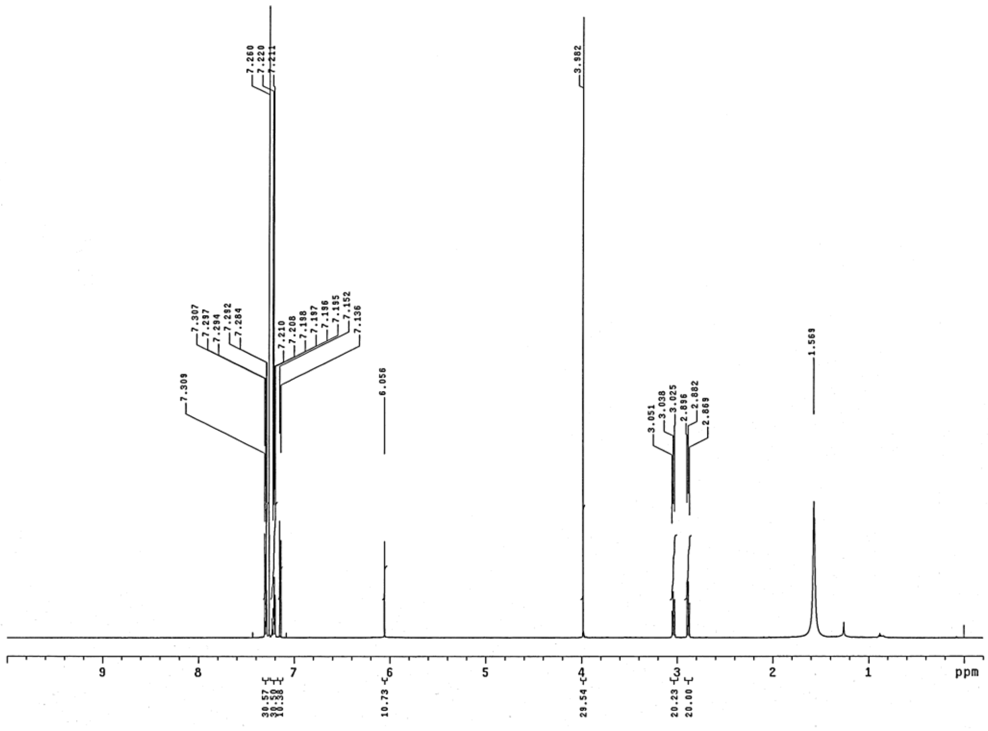

Figure S27. ^{1}H-NMR spectrum ($CDCl_3$, 600 MHz) of **4**.

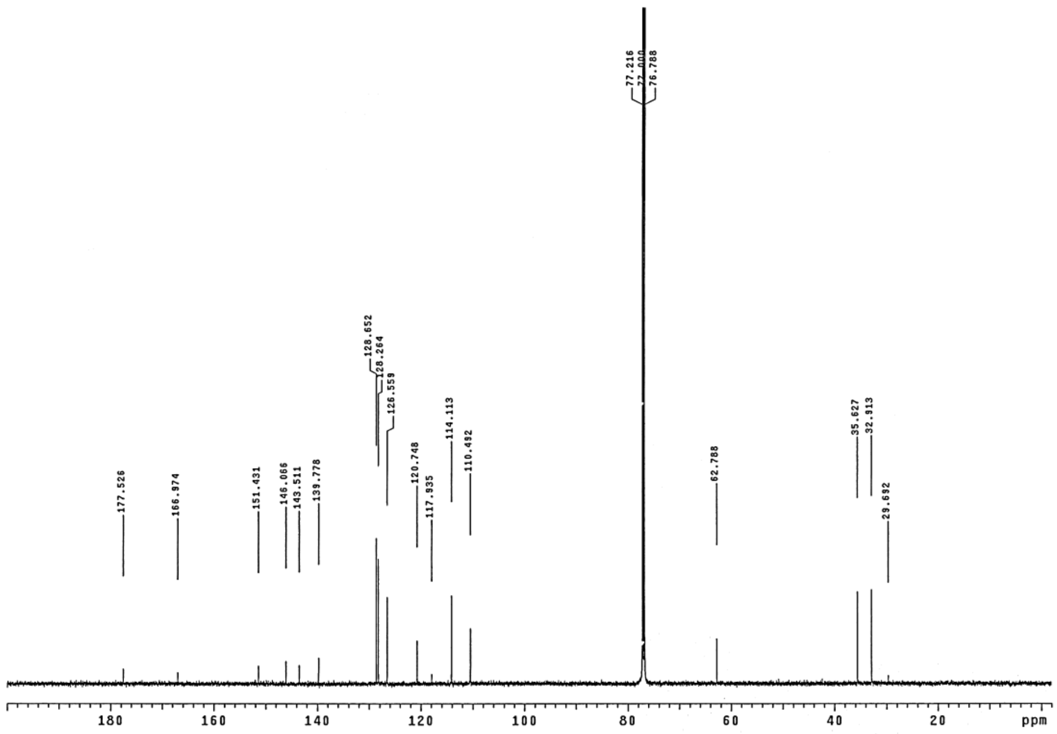

Figure S28. ^{13}C-NMR spectrum of **4** ($CDCl_3$, 150 MHz)

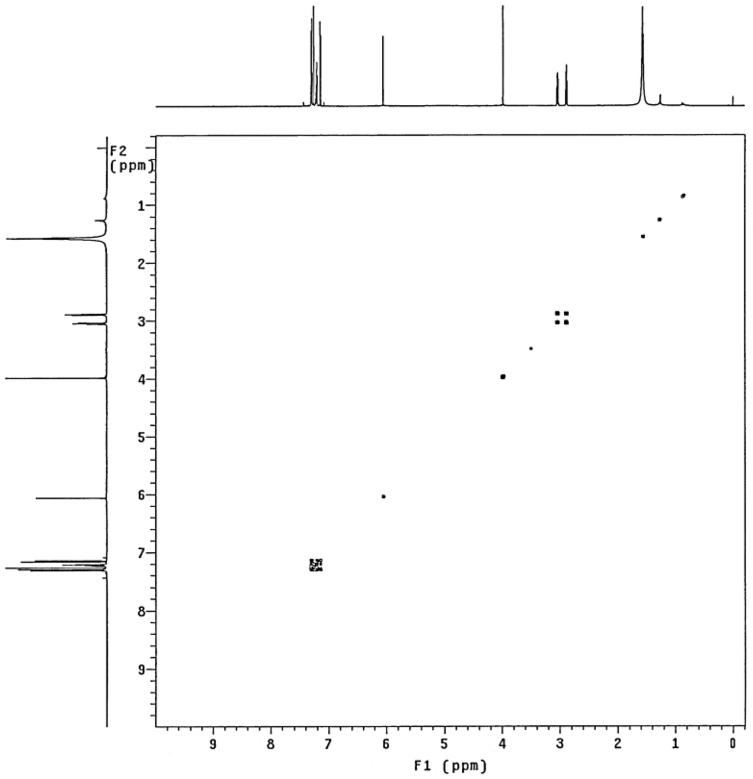

Figure S29. ^{1}H–^{1}H COSY spectrum of **4**.

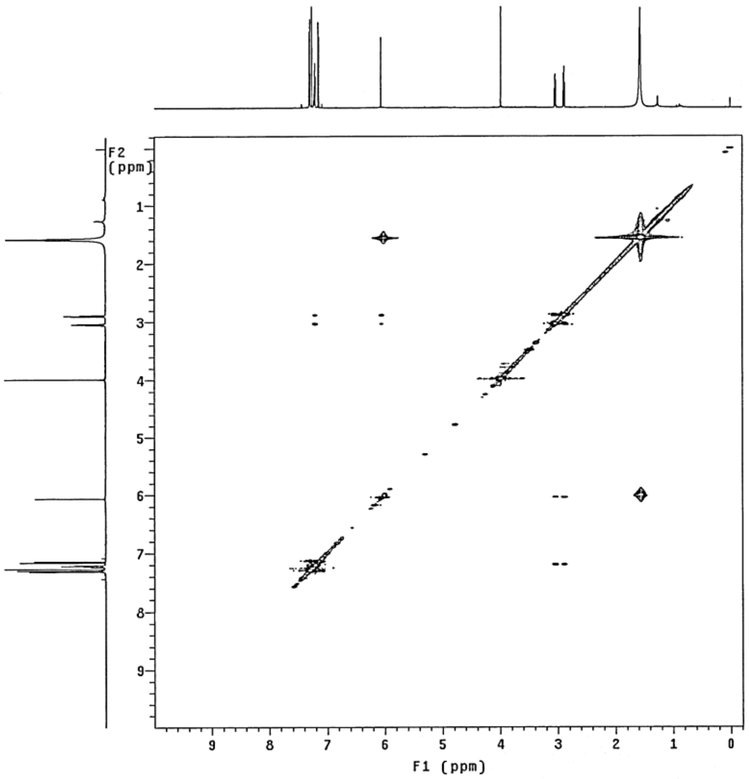

Figure S30. NOESY spectrum of **4**.

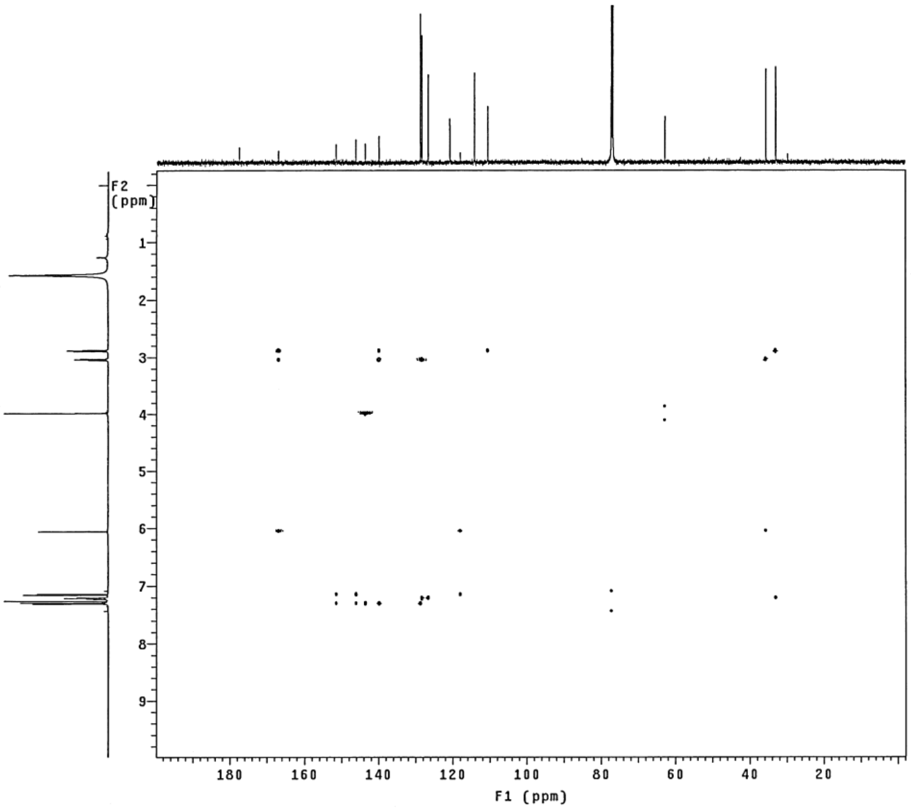

Figure S31. HMBC spectrum of **4**.

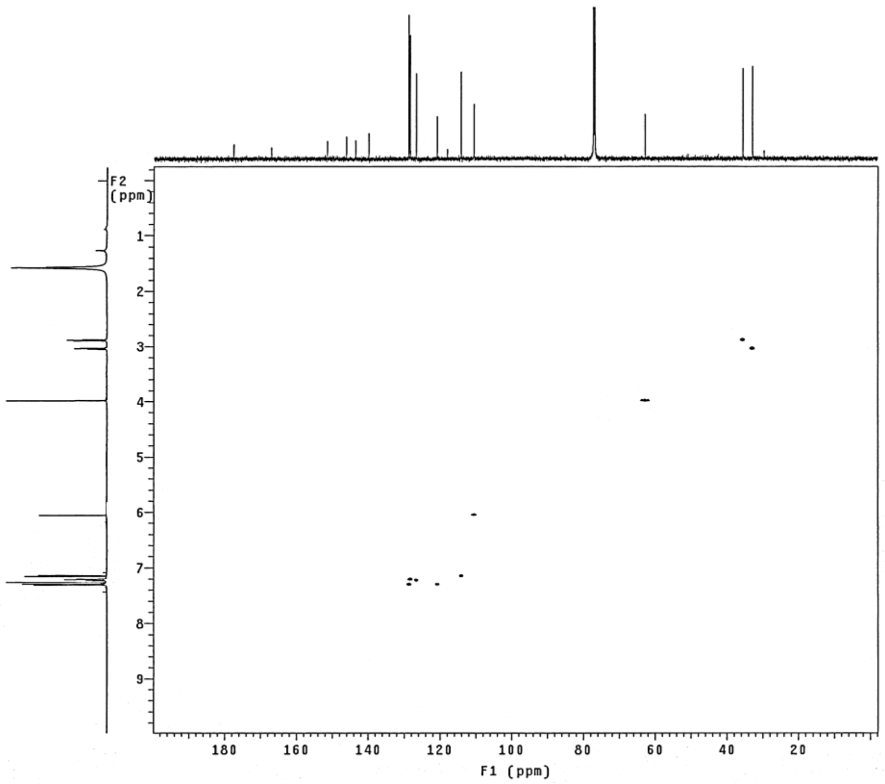

Figure S32. HSQC spectrum of **4**.

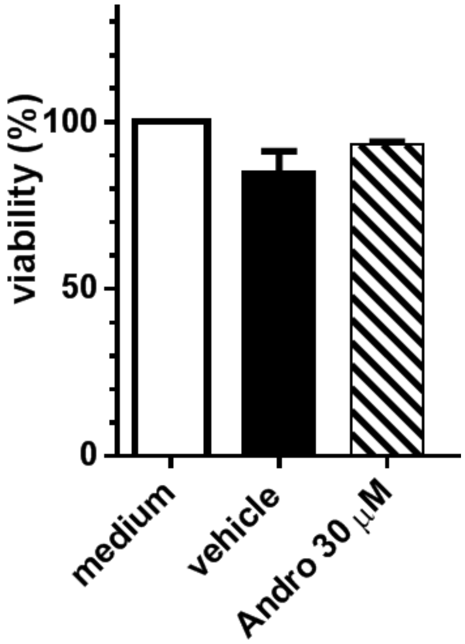

Figure S33. The data of cell viability after andrographolide treatment.
